# Decline of a distinct coral reef holobiont community under ocean acidification

**DOI:** 10.1186/s40168-023-01683-y

**Published:** 2024-04-17

**Authors:** Jake Williams, Nathalie Pettorelli, Aaron C. Hartmann, Robert A. Quinn, Laetitia Plaisance, Michael O’Mahoney, Chris P. Meyer, Katharina E. Fabricius, Nancy Knowlton, Emma Ransome

**Affiliations:** 1 Georgina Mace Centre for the Living Planet, Department of Life Sciences, Imperial College London, Buckhurst Road, Ascot, SL5 7PY UK; 2 Institute of Zoology, Zoological Society of London, Regent’s Park, London, NW1 4RY UK; 3 Department of Organismic and Evolutionary Biology, Harvard University, Cambridge, MA USA; 4 Department of Biochemistry and Molecular Biology, Michigan State University, East Lansing, MI 48824 USA; 5 Laboratoire Evolution Et Diversité Biologique, CNRS/UPS, Toulouse, France; 6 National Museum of Natural History, Smithsonian Institution, Washington, DC 20013 USA; 7 Australian Institute of Marine Science, Townsville, Queensland Australia

**Keywords:** Microbialisation, Ocean acidification, Multiomics, Holobiont, Autonomous Reef Monitoring Structures, Ecosystem collapse

## Abstract

**Background:**

Microbes play vital roles across coral reefs both in the environment and inside and upon macrobes (holobionts), where they support critical functions such as nutrition and immune system modulation. These roles highlight the potential ecosystem-level importance of microbes, yet most knowledge of microbial functions on reefs is derived from a small set of holobionts such as corals and sponges. Declining seawater pH — an important global coral reef stressor — can cause ecosystem-level change on coral reefs, providing an opportunity to study the role of microbes at this scale. We use an in situ experimental approach to test the hypothesis that under such ocean acidification (OA), known shifts among macrobe trophic and functional groups may drive a general ecosystem-level response extending across macrobes and microbes, leading to reduced distinctness between the benthic holobiont community microbiome and the environmental microbiome.

**Results:**

We test this hypothesis using genetic and chemical data from benthic coral reef community holobionts sampled across a pH gradient from CO_2_ seeps in Papua New Guinea. We find support for our hypothesis; under OA, the microbiome and metabolome of the benthic holobiont community become less compositionally distinct from the sediment microbiome and metabolome, suggesting that benthic macrobe communities are colonised by environmental microbes to a higher degree under OA conditions. We also find a simplification and homogenisation of the benthic photosynthetic community, and an increased abundance of fleshy macroalgae, consistent with previously observed reef microbialisation.

**Conclusions:**

We demonstrate a novel structural shift in coral reefs involving macrobes and microbes: that the microbiome of the benthic holobiont community becomes less distinct from the sediment microbiome under OA. Our findings suggest that microbialisation and the disruption of macrobe trophic networks are interwoven general responses to environmental stress, pointing towards a universal, undesirable, and measurable form of ecosystem change.

Video Abstract

**Supplementary Information:**

The online version contains supplementary material available at 10.1186/s40168-023-01683-y.

## Background

Increased atmospheric greenhouse gas concentrations are leading to increased partial pressure of CO_2_ (pCO_2_) and reduced pH in the surface water of the oceans, which have absorbed 25% of all anthropogenic CO_2_ to date [1]. The impacts of this phenomenon, known as ocean acidification (OA) [2], are predicted to be particularly severe for coral reefs due to declines in net calcification by organisms, which impacts reef structure and diversity [3]. Severe impacts on coral reefs are concerning as these ecosystems host vast biodiversity and provide significant ecosystem services to humanity (e.g. coastal protection, food security, and new medicines), and other significant terrestrial and pelagic ocean ecosystems are also directly dependent on their functioning [4]. Being able to predict how coral ecosystems change under OA, and what this means for their ability to continue providing ecosystem services to society, is therefore a global environmental and social priority.

Microbes play important roles on coral reefs, from carrying out nutrient cycling, which contributes to ecosystem productivity in nutrient poor waters [5], to their roles in immunity and defence for a wide range of reef invertebrates, including cnidarians, sponges, molluscs, and echinoderms [6–9]. These interactions between macrobes and microbes are known to shift in response to environmental change, such as OA. Such shifts include changing microbial associations with particular macrobes, such as corals [10, 11] and sponges [12], and changes in the functional profile of specific microbiomes, such as alterations in nitrogen (N_2_) fixation by coral-associated bacteria [13] and shifting metabolic activity of free-living bacteria in the water column [14]. However, the traditional focus of research on individual macrobes and their microbiomes (holobionts) makes it challenging to scale up our understanding to ecosystem-level shifts in macrobe-microbe interactions, which, though often overlooked, can play a significant role in driving ecosystem-level change [11, 15, 16].

Ecosystem-level impacts of OA are expected to result from interaction-mediated changes at the community level, driven by different physiochemical effects of OA at the level of organism metabolism [2, 17]. Well-established organism-level effects of OA include benefits to fleshy algae and other photosynthetic organisms from the resource effect of increased pCO_2_ [2, 18], which can result in enhanced net dissolved organic carbon (DOC) release [19]. In contrast, calcifiers suffer from increased costs of calcification due to reduced pH [2]. These differentiated organism-level impacts can shift ecological interactions between taxa [2, 17], with cascading effects on energy flows through an ecosystem via altered nutrition and metabolism (i.e. altered trophodynamics [20, 21]). Such indirect cascading effects can impact entire trophic networks [22], including macrobe-microbe trophic interactions [23]. However, our understanding of the indirect effects of ocean acidification on coral reefs remains limited [24].

Here, we propose that a previously observed ecosystem-level impact of specific stressors on coral reefs — microbialisation — may be generalisable to OA. Microbialisation refers to an increase in microbial biomass resulting from a reallocation of energy from macrobes to microbes [23, 25]. On coral reefs, the proximal causes of microbialisation have been proposed to be overfishing and eutrophication, which facilitate the enhanced growth of fleshy algae and cause an increased release of dissolved organic carbon (DOC) [26]. Elevated DOC has been proposed to increase microbial biomass and disease (the DDAM (DOC, disease, algae, microbes) positive feedback loop) [26].

We build on a commonality between proposed organism-level mechanisms of microbialisation, namely that macrobe communities are expected to be more vulnerable to stressors than microbial communities [26, 27], an observation both supported in general [28] and in the case of OA in particular [29]. This greater vulnerability of macrobes should lead to declines in some benthic taxa, such as calcified algae, soft and hard corals, sponges [18], and calcified grazers (e.g. gastropods [30]), already documented with OA, and to the disruption of associated macrobe trophic pathways. Examples of these trophic pathways include calcified grazers consuming algal communities (which controls algal proliferation) [31], and sponges removing vast quantities of dissolved organic matter (DOM) from the water column and converting it into food for higher trophic levels (e.g. polychaetes and brittle stars) via the sponge loop [32]. With declines in benthic taxa, some of the free energy cycled through such pathways can become available to less impacted taxa, such as environmental microbes [20, 33], and is expected to drive the ecological release of such taxa in both density and niche expansion [34].

We hypothesise that we will observe a decline in the compositional and functional distinctness between the benthic holobiont community microbiome and the environmental sediment-dwelling microbiome as a result of microbialisation occurring under OA. Almost all macrobes are holobionts with a symbiotic microbiome [35, 36], and therefore, microbialisation has the potential to impact the microbiome of the entire holobiont community (recently referred to as the eco-holobiont [37]). The process of microbialisation should result in decreased compositional and functional distinctness between the benthic holobiont community microbiome and the sediment microbiome through two mechanisms. Firstly, direct impacts on macrobes may alter host metabolism and reduce the resources or habitat available to the holobiont community microbiome, leading to opportunistic environmental microbes displacing holobiont-specialised microbes (e.g. [38]). Secondly, increased abundances of environmental microbes and increased microbe trophic interactions with macrobes (expected due to the loss of macrobe competitors [39]) should lead to increased opportunities for colonisation of the holobiome by environmental microbes [40], including those in the sediment microbiome.

To test whether this decline in the distinctness of the benthic holobiont community microbiome occurs, we generated a unique multiomic dataset from autonomous reef monitoring structures (ARMS) deployed on a natural OA gradient caused by CO_2_ seeps. ARMS are three-dimensional, artificial settlement structures designed to mimic the structural complexity of coral reef environments, which are increasingly used to monitor coral reefs across the globe (e.g. [41–43]). They enable the non-destructive and standardised sampling of a large proportion of reef diversity that is often not studied, including algae and cryptic benthic invertebrates such as sponges, cnidarians, bryozoans, and annelids [44], alongside their associated microbes, and sediment-dwelling environmental microbes. ARMS allow us to investigate OA using a holistic microbial ecosystem approach that integrates across scales from individual microbes and benthic holobionts, to neighbouring holobionts that, in turn, interact with and influence successively larger and more complex communities. We studied the effects of OA using an in situ experimental approach at a location with naturally occurring CO_2_ seeps that produce pH and pCO_2_ gradients. These seeps have been intensively studied because they can help predict future ocean conditions under OA [45].

Multiomics, in this case metabarcoding and metabolomics, provides a powerful toolkit to investigate the effects of stressors across entire communities. Metabarcoding provides data on genetic community composition and diversity (i.e. compositional metrics) [46], while metabolomics provides comparable data on biochemical composition and diversity of the metabolome (i.e. functional metrics) [47]. We first confirm that the expected photosynthetic community shifts take place (as previously documented [18]), consistent with microbialisation. We then analyse the ecosystem-level effects of OA by comparing the distinctness of the sediment microbiome to: (1) the benthic holobiont community microbiome and (2) individual sponge microbiomes. We expected OA to cause a decline in distinctness as a result of ecosystem microbialisation.

## Materials and methods

### Experimental design and sampling

**Fig. 1:**
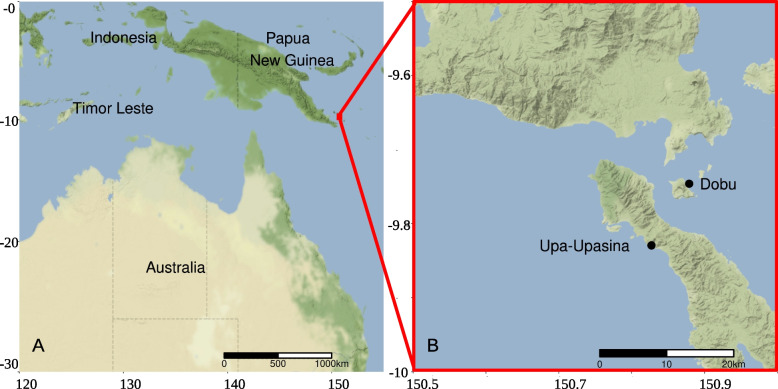
Location of the study localities, Dobu and Upa-Upasina, in Milne Bay Province, Papua New Guinea (**A** & **B**)

This study was carried out at CO_2_ seeps and adjacent control sites in Milne Bay Province, Papua New Guinea (Fig. 1), located at 9° s latitude in the heart of the Coral Triangle. The two studied seep localities (Upa-Upasina and Dobu) are located along an active tectonic fault where > 99% CO_2_ gas has been streaming though the reef substrata at ambient temperature (28.6–29.7 °C) for at least 100 years and probably much longer [18]. The reefs surrounding the seeps are under low anthropogenic pressure and have been used to study ocean acidification for the last decade (e.g. [18, 48]). The six study sites (two localities, each with three pH levels) exhibit similar geomorphology, temperature and salinity, but contrasting pCO_2_ and pH [18]. Water temperature, pH, salinity, and pressure at the study sites have been monitored regularly (2010–2016), making this an ideal location to study the isolated impacts of OA.

**Fig. 2:**
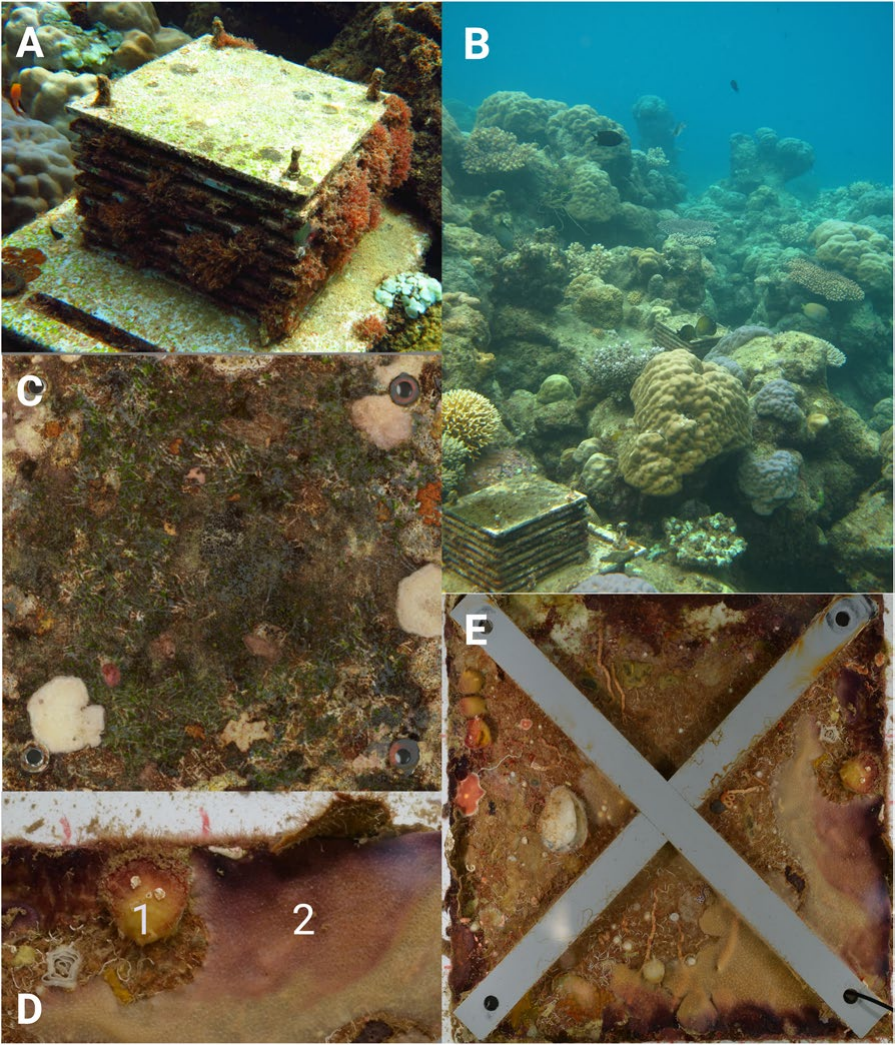
Images of Autonomous Reef Monitoring Structures (ARMS) and ARMS plates. Images show an ARMS in situ (**A**) and nestled within the reef after 2.5 years of deployment (**B**). A light-exposed ARMS top plate, from which the benthic photosynthetic community was sampled, can be seen in (**C**). A *Tethya* sp. sponge (1) and a *Halisarca* sp. sponge (2), commonly observed on recovered ARMS, can be seen in (**D**). An internal light-limited ARMS plate with crossbars, which create sheltered conditions and mimic the natural reef, can be seen in (**E**)

In April, 2012, eighteen autonomous reef monitoring structures (ARMS; Fig. 2A) were deployed at 3 m depth adjacent to coral reefs at 3 pH levels at each of the 2 localities (mean pH: control 7.99 & 8.01, medium 7.85 at both localities, and low 7.64 & 7.75; *n* = 3 per pH level [48]). ARMS were collected from the seafloor (Fig. 2B) after 31 months, in November 2014. A 106-μm nitex-lined crate was placed over each ARMS on the seafloor, and they were together returned to the surface, after which each ARMS was placed in an individual holding tank with 45-μm filtered aerated seawater. ARMS were then transported to shore where they were sampled rapidly to minimise molecular and chemical degradation; transportation time of ARMS was < 20 min, and processing of each sample type was completed within 1.5 h.

### Sample extraction and multiomics

The standard ARMS processing protocol [44] was modified to test our specific hypothesis. From each ARMS unit, five fractions were collected: the benthic photosynthetic community, the benthic holobiont community, the sediment, *Halisarca* sp. sponge, and *Tethya* sp. sponge. To do this, ARMS were removed from their holding tanks, and the 9 plates (17 plate surfaces as there is no accessible bottom surface to the bottom plate) were separated and rinsed to dislodge loosely attached organisms. The water and previously trapped sediment in the holding tank were retained.

First, the benthic photosynthetic community was sampled by randomly subsampling (4 × 1 cm^2^) the top surface of the top ARMS plate (e.g. Figure 2C), which resembles the algal community found on exposed rocky substrates (e.g. macroalgae, algal turf, calcified algae, and cyanobacterial mats). Second, the two sponge fractions (*Halisarca* sp. and *Tethya* sp., e.g. Figure 2D) were generated by sampling morphologically identified sponges from the internal plates. Both are low-microbial abundance sponges [49, 50], and *Tethya* sp. was only found at Upa-Upasina. Thirdly, the benthic holobiont community fraction was generated by scraping, and blending the scrapings from all 17 surfaces, including the remainder of the light-exposed top surface of the top plate. While this fraction will include both photosynthetic and non-photosynthetic organisms, the shaded plate surfaces constitute ~ 16 × the area of the light-exposed top surface of the top plate. The most abundant phyla found in this fraction on other reefs around the world are fairly consistent and include the Porifera, Cnidaria, Bryozoa, Chordata (Ascidiacea), and Annelida [42–44]. An example of an ARMS plate from which this fraction was collected can be seen in Fig. 2E. This homogenised bulk sample was then subsampled (50 ml). Finally, the sediment fraction was generated by passing the water and sediment from the holding tank through a 500 μm and then a 100-μm sieve and collecting the material which did not pass through the 100-μm sieve. This drained sediment sample was subsampled (10 g). This fraction was therefore primarily composed of sediment, microbes, including free-living sediment dwelling microbes (e.g. bacteria) and microplankton (e.g. single-celled algae such as diatoms and dinoflagellates).

From each ARMS unit, each fraction was split in two; one-half was snap frozen for metabolomic analysis by being dropped in liquid nitrogen in a dry shipper, and the other was placed in RNA later for metabarcoding. All samples were returned to the Smithsonian National Museum of Natural History (Washington, DC, USA) in a liquid nitrogen dry shipper. Total DNA was extracted from 10 g of the benthic holobiont community samples and 5 g of the sediment samples using a MO-BIO PowerMax Soil DNA Isolation Kit according to the manufacturer’s protocol with the addition of 400 μg/ml proteinase K and an overnight lysis step at 56 °C and 200 rpm. The benthic photosynthetic community and sponge subsamples were extracted with the DNeasy Blood and Tissue Kit (Qiagen), according to the manufacturer’s instructions. All DNA extracts were purified using MO-BIO PowerClean DNA Clean-Up Kits, quantified Qubit dsDNA HS Kit, run on an agarose gel, and DNA quality investigated using ImageJ software. All sample types are known to contain relatively high bacterial biomass, and thus, we do not expect contamination from the lab environment or equipment to be a major issue in DNA libraries. However, extraction and PCR amplification controls were included for all sample types; these were all negative and so were not sequenced. Each sample was analysed with 16S rRNA gene metabarcoding (for the microbe community) and mass spectrometry (for metabolomics). All 16S rRNA gene libraries were prepared for sequencing using the original Earth Microbiome Project protocol using primers 515 F and 806 R, which are designed to amplify prokaryotes (Bacteria and Archaea) [51]. To investigate the benthic photosynthetic community, 23S rRNA gene libraries were also prepared using the protocol described by Marcelino and Verbruggen [52] using a two-step PCR procedure that first amplifies the gene fragment followed by ligation of the barcoded Illumina adaptors to the amplicons in a second PCR reaction; this protocol is designed to target both eukaryotic algae and cyanobacteria.

Metabolites were extracted from all fractions in 70% methanol [47]. The 70% methanol extraction was chosen to select for slightly polar molecules, encompassing a broad range of the chemosphere [53]. Metabolites were separated and identified via liquid chromatography-tandem mass spectrometry using a Bruker Daltonics Maxis qTOF mass spectrometer equipped with a standard electrospray ionisation source according to the methods of Quinn and colleagues [53]⁠. Briefly, the mobile phase was pumped through a Kinetex 2.6 μm C18 (30 × 2.10 mm) ultra-performance liquid chromatography (UPLC) column for a 15-min run. The resulting LC–MS/MS data files were processed through the MZmine2 workflow. The subsequent metabolite feature table was then processed through the GNPS feature-based molecular networking workflow with the default parameters, except that a minimum cosine of 0.65 and a minimum matched peaks of 4 were used for network construction.

### Bioinformatics

A bioinformatic pipeline was implemented in R for the 16S and 23S rRNA gene libraries. Amplicon sequence variants (ASVs) were generated from raw sequencing data using the Divisive Amplicon Denoising Algorithm (DADA2 v1.24.0 [54]). Reads were quality filtered to maintain Q30 scores while maintaining at least 50 base pair overlap and removing any base pair below Q2 [55]. Default maxEE (2) and truncQ (2) parameters were used, 16S rRNA gene sequences were truncated at a length of 150 base pairs on both strands, 23S rRNA gene sequences had the first 20 base pairs (nonbiological primers) trimmed from both strands and were truncated at a length of 249 base pairs on the forward strand and 212 base pairs on the reverse strand (see Table S1 for numbers of sequences passing denoising steps). Taxonomy was assigned using the DECIPHER v2.24.0 R package [56] — which has been shown to have higher accuracy than popular classifiers including BLAST and the RDP classifier [56] — and the GTDB (16S rRNA gene [57]) and microgreen (23S r RNA gene [58]) databases. 16S rRNA gene ASVs identified as plastids were subsequently removed. Samples were not rarefied as part of bioinformatic processing [59] but only when required for specific statistical analyses (see ASV-level Shannon diversity below).

### Statistical analyses

PERMANOVAs were performed for each fraction separately, with the 16S rRNA gene sequencing and metabolomic data subdivided into community fractions (benthic photosynthetic community, benthic holobiont community and sediment) and organism fractions (*Halisarca* sp. and *Tethya* sp. sponges), resulting in 10 PERMANOVAs (Table S2). Prior to fitting PERMANOVAs, a multivariate analogue of Levene’s test for homogeneity of variances (betadisper) was applied to ensure PERMANOVA tests could be applied. PERMANOVAs fit locality and ordinal pH as explanatory variables, and locality was treated as a blocking factor, except in the case of the *Tethya* sp. sponge which was only found at one locality and so was fit with pH as the only explanatory variable. An eleventh PERMANOVA was run on the 23S rRNA gene data, following the same approach. Bonferroni corrections were applied to all *p*-values obtained from the PERMANOVAs to account for multiple testing. All PERMANOVAs and supporting NMDS visualisations were based on Morisita dissimilarities between samples, as they have been shown to be most reliable in the case of under sampling [60].⁠

Total ASV richness and phylum level Shannon diversity, each accounting for unobserved ASVs, were estimated for each metabarcoding sample using a breakaway model [61] and a DivNet model, treating all samples as independent observations, respectively [62]. The estimated richness and estimated Shannon diversity of all metabarcoding samples were then modelled using a single betta hierarchical mixed model for each metric (including all fractions). This modelling approach was chosen to account for explanatory variables, richness variance, and richness estimation error [61]. Fraction, ordinal pH, and the interaction of fraction and pH were included as fixed effects and locality as a random effect. Compound richness and Shannon diversity were calculated from untransformed data for metabolomic samples from all fractions. Each metric was modelled with a single linear mixed model. Fraction, pH, and the interaction of fraction and ordinal pH were included as fixed effects and locality as a random effect. In addition, ASV level Shannon diversity was calculated (no statistical estimation procedure was applied) for all samples rarefied to even depth (*n* = 50,000) to test the sensitivity of the results found for phylum level Shannon diversity.

The change in abundance of phyla and metabolites with OA was analysed using DeSeq2 differential abundance analysis with a negative binomial distribution [63]⁠. Differential abundance and dispersions were calculated for each community fraction (benthic photosynthetic community, benthic holobiont community, and sediment) and multiomic analysis type separately using a DESeq2 design formula with variables of locality and pH. This enabled change within each community fraction to be examined. However, abundance and dispersions were calculated for both sponge fractions together using a design formula with variables of species, locality, and pH. This enabled shared change occurring across sponges under OA to be examined. Wald significance tests were conducted for changes in differential abundance under OA, with a parametric fit of dispersions [63].

Microbiome/metabolome distinctness was calculated for each ARMS as the proportion of unique sequences found within the benthic holobiont community microbiome/metabolome which were not also found in the sediment microbiome/metabolome. Microbiome/metabolome distinctness was modelled using a linear mixed model with ordinal pH as a fixed effect and locality as a random effect. A likelihood ratio test was used to infer the significance of ordinal pH as a fixed effect. Note that this analysis was not conducted for the benthic photosynthetic community as we are testing whether distinctness is reduced for the general community of macro-organisms, and the benthic holobiont community is a more general sample including both photosynthetic and non-photosynthetic organisms. The same approach was taken to calculate and model microbiome/metabolome distinctness for individual holobionts with the additional random effects of (i) ARMS identity (nested within locality), as multiple individual holobionts were collected from the same ARMS, and (ii) sponge species.

Benthic holobiont community microbiome distinctness from the sediment microbiome was also modelled with a modified mixture Sloan neutral community model (MSNCM [64]). This additional modelling approach captures the contribution of each of the sediment and benthic holobiont community microbiome metacommunities to the composition of benthic holobiont community microbiomes from individual ARMS, thus providing an alternative abundance-based test of whether benthic holobiont community microbiomes become more distinct from the sediment microbiome under OA. The original Sloan neutral community model describes the frequency of occurrence of ASVs in a community as a function of their abundance in the metacommunity, with a single free parameter (m: migration) which can be interpreted as the probability of neutral dispersal or alternatively inverse dispersal limitation. The MSNCM used here models ASV frequency in sampled benthic holobiont community microbiomes from each pH regime as a function of its abundance in two metacommunities: (1) all benthic holobiont community microbiomes from the same pH regime as the sample and (2) all sediment microbiomes from the same pH regime as the sample. Each metacommunity is fit with its own migration/inverse dispersal limitation parameter (mholo, menv), and a mixture parameter (mix) is fit describing the contribution of each metacommunity. The model is fit to samples from each pH regime separately using non-linear least-squares fitting as detailed in Burns et al. [65]⁠.

All statistical analyses were conducted in R (version 4.2.1 [66])⁠; specific packages used were as follows: phyloseq v1.40.0 [67] for data manipulation, vegan v2.6–4 [68] for PERMANOVA and betadisper, breakaway v4.8.2 [61] and DivNet v.0.4.0 [62] for diversity estimation and modelling, lme4 v1.1–31 [69] and MuMIn v1.47.1 [70] for generalised linear mixed models, DESeq2 v1.36.0 for differential abundance analysis [63], and minpack.lm v1.2–2 [71] and Hmisc v4.7–1 [72] for non-linear least-squares modelling.

## Results

### Genetic diversity and composition

Five fractions were generated for analysis: benthic photosynthetic community, benthic holobiont community, sediment, *Tethya* sp., and *Halisarca* sp. Benthic photosynthetic communities were dominated by red algae (Rhodophyta), brown algae (Ochrophyta), and Cyanobacteria. Benthic holobiont communities were visually dominated by Porifera, Chordata, Bryozoa, Annelida, Arthropoda, and Mollusca. Benthic holobiont community microbiomes were dominated by Proteobacteria, unclassified Bacteria, and Cyanobacteria. Benthic photosynthetic community microbiomes were dominated by Proteobacteria and Cyanobacteria, followed by Firmicutes, Bacteroidota, and unclassified Bacteria. Sediment microbiomes were dominated by Proteobacteria, unclassified Bacteria, Bacteroidota, and Planctomycetota. Sponge microbiomes were dominated by Proteobacteria; unclassified Bacteria ASVs were also highly abundant in *Tethya* sp. samples. See Figure S1.

**Fig. 3:**
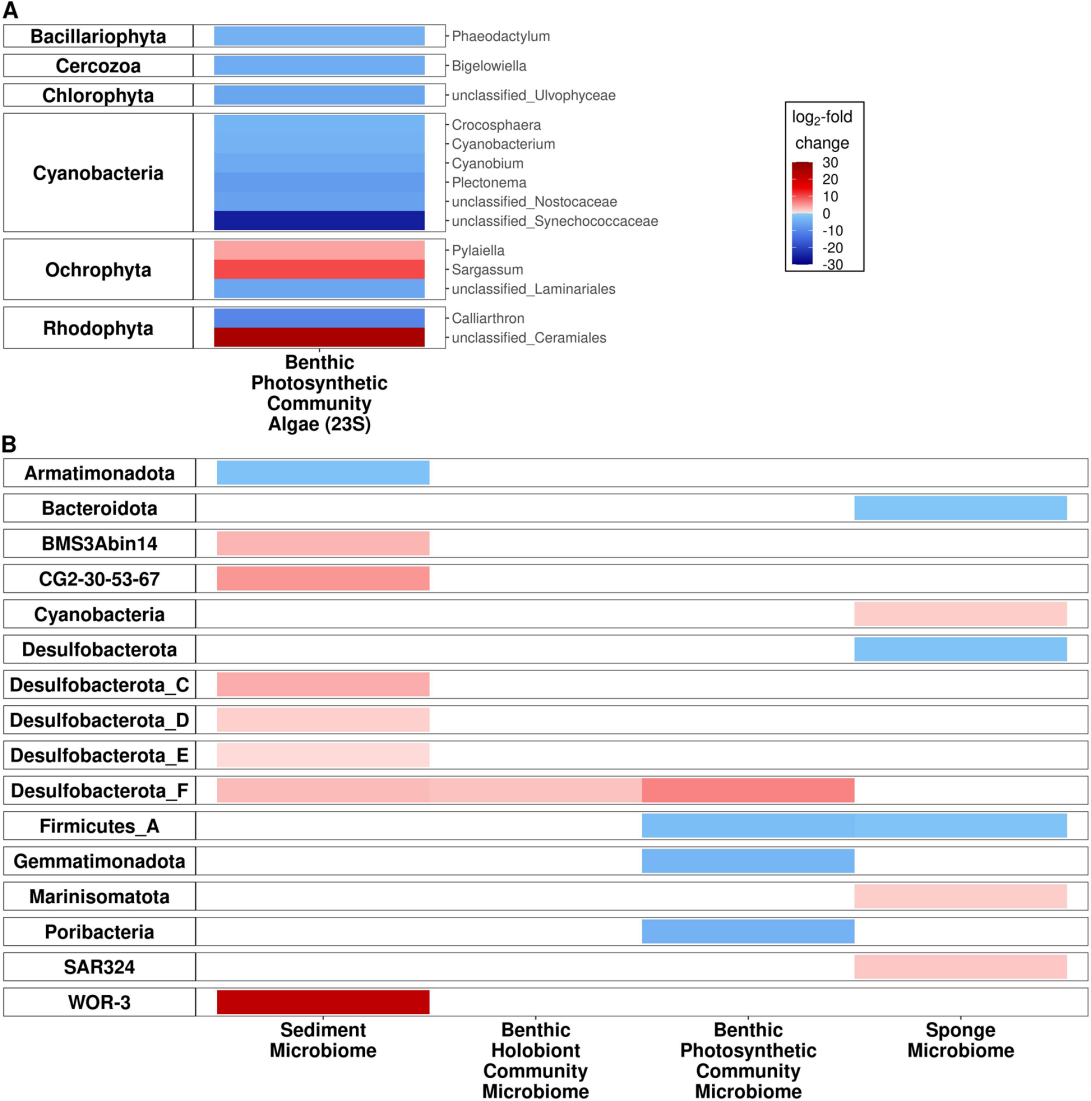
Heat maps of significant differential abundance of phyla with decreasing pH. Significant change in differential abundance of algal phyla (23S rRNA gene) with decreasing pH are seen in (**A**); algal phyla are shown on the left, and families are shown on the right. Algal taxonomy was assigned using the microgreen database [58]. Significant change in differential abundance of microbial phyla (16S rRNA gene) with decreasing pH are seen in (**B**); bacterial phyla are shown on the left. Microbial taxonomy was assigned using the GTDB taxonomy [57]

Fifteen 23S rRNA gene metabarcoding libraries were generated across the pH gradient, to confirm the expected effect of OA on the benthic photosynthetic communities. The composition of the benthic photosynthetic community differed significantly by pH (*F* = 5.5, *p* < 0.05), with significant declines in phylum Shannon diversity (95% *CI* [–0.76, –0.55], *p* < 0.05) and ASV Shannon diversity (95% CI [–2.27, –0.33], *p* < 0.05) at lower pH. Lower pH was associated with significantly increased differential abundance of the dominant phylum Ochrophyta (of which 99.7% of reads were from the class Phaeophyceae, and 71.4% were from the genus *Sargassum*; Fig. 3; Figure S1).

Ninety-four 16S rRNA metabarcoding libraries were generated across the pH gradient, from 18 ARMS. Each fraction (benthic photosynthetic community microbiome, sediment microbiome, benthic holobiont community microbiome, and sponge [*Tethya* sp. and *Halisarca* sp.] microbiomes) had between 15 and 30 samples (Table 1), which in total produced 55,348 ASVs (*n* = 94). Eighty bacterial phyla were identified, with 77.7% of reads identified to the level of phylum.

**Table 1:** Summary table of all 16S rRNA gene and metabolomic data generated

	**Metabarcoding — 16S rRNA gene**					**Metabolomics — MeOH solvent extraction**				
	**Community fractions**			**Single holobiont fractions**	**All 16s rRNA gene**	**Community fractions**			**Single holobiont fractions**	All metabolomes
	Sediment	Benthic photosynthetic community	Benthic holobiont community	Sponge		Sediment	Benthic photosynthetic community	Benthic holobiont community	Sponge	
**Samples (control, medium, low)**	17 (6; 5; 6)	15 (5; 5; 5)	15 (6; 4; 5)	47 (18; 13; 16)	94 (35; 27; 32)	18 (6; 6; 6)	19 (7; 6; 6)	18 (6; 6; 6)	52 (19; 16; 17)	107 (38; 34; 35)
**Median ASVs/compounds per sample (mean)**	11,553 (11,437)	2497 (2575)	7414 (7558)	1756 (1902)	2596 (4685)	192.5 (191)	176 (169)	164 (172)	172 (181)	178 (179)
**Median reads/biomolecules per sample (mean)**	226,704 (223,047)	191,380 (189,806)	228,828 (231,210)	179,696 (188,960)	199,515 (202,050)	217,272,202 (222,010,376)	164,003,665 (184,941,718)	149,861,437 (163,084,039)	200,557,415 (208,791,822)	189,723,756 (199,091,279)
**ASVs identified at phylum/compounds identified (percent reads/molecules)**	70.8 (87.8)	76.9 (87.4)	74.1 (73.4)	74.9 (71.9)	69 (77.7)	5.04 (4.27)	4.77 (1.57)	6 (3.29)	7.79 (8.8)	4.62 (6)
**Median phyla per sample (mean)**	57 (56.6)	29 (30.5)	46 (45.9)	38 (37.8)	40 (41.4)	NA	NA	NA	NA	NA
**Median ASV/metabolomic richness per sample (mean)**	11,985 (11,920)	2750 (2802)	7909 (8006)	1984 (2098)	2991 (5071)	192 (191)	176 (169)	164 (172)	172 (181)	178 (179)
**Median ASV/metabolomic Shannon diversity per sample (mean)**	2.12 (2.13)	1.4 (1.39)	2.02 (2.02)	0.971 (0.92)	1.16 (1.39)	2.83 (2.81)	2.99 (2.83)	2.76 (2.76)	2.96 (3.04)	2.87 (2.92)

Forty-seven 16S rRNA gene metabarcoding libraries were generated across the benthic photosynthetic community, benthic holobiont community, and sediment microbiomes (*n* = 15, 17, 15, respectively). All community microbiomes were significantly compositional different at lower pH (control pH compared with medium and low pH as an ordinal variable): benthic photosynthetic community microbiome (*F* = 2.3, *p* < 0.05), sediment microbiome (*F* = 3.9, *p* < 0.05), and benthic holobiont community microbiome (*F* = 3.0, *p* < 0.05; Table S2B; Figure S2). There was no significant effect of pH on richness for any community microbiome (Table S3). Phylum and ASV level Shannon diversity was significantly lower in the benthic photosynthetic community microbiome at lower pH (95% *CI* [− 0.55, − 0.09], *p* < 0.05; Table S4; Figure S3 and 95% *CI* [− 2.5, − 0.64], *p* < 0.05; Table S5; Figure S3, respectively). Please see Table S6 for a summary of all significant 16S rRNA patterns. Decreased pH was associated with significant differences in the abundance of the following phyla: increased *WOR-3* and Desulfobacterota alongside decreased Armatimonadota in the sediment microbiome, increased Desulfobacterota in the benthic holobiont community microbiome, and increased Desulfobacterota alongside decreased Poribacteria, Gemmatimonadota, and Firmicutes in the benthic photosynthetic community microbiome (Fig. 3).

Forty-seven 16S rRNA gene metabarcoding libraries were generated from the microbiomes of the two sponge species: 17 from *Halisarca* sp. individuals and 30 from *Tethya* sp. individuals. Microbiome composition was only significantly different across the pH gradient for *Tethya* sp. sponges (*F* = 5.9, *p* < 0.05; Table S2; Figure S2). While there was no significant effect of pH on ASV richness or ASV level Shannon diversity, reduced pH was associated with significantly lower phylum level Shannon diversity in *Tethya* sp. sponge microbiomes (95% *CI* [− 0.44, − 0.12], *p* < 0.05), but no such effect was found in *Halisarca* sp. sponge microbiomes (Table S4; Figure S3; see Table S6 for a summary of all significant 16S rRNA patterns). At the phyla level, lower pH was associated with significantly higher abundances of Marinisomatota, *SAR324*, and Cyanobacteria and significantly lower abundances of Firmicutes, Desulfobacterota, and Bacteroidota (Fig. 3) in both sponge species microbiomes.

### Biochemical diversity and composition

One-hundred and seven metabolome libraries were analysed from the same 18 ARMS. Each fraction (benthic photosynthetic community metabolome; benthic holobiont community metabolome, sediment metabolome, and sponge metabolomes) had between 18 and 34 samples across the pH gradient (Table 1). These samples produced 1211 compounds, of which 4.62% were identified, representing 6% of all molecules.

**Fig. 4:**
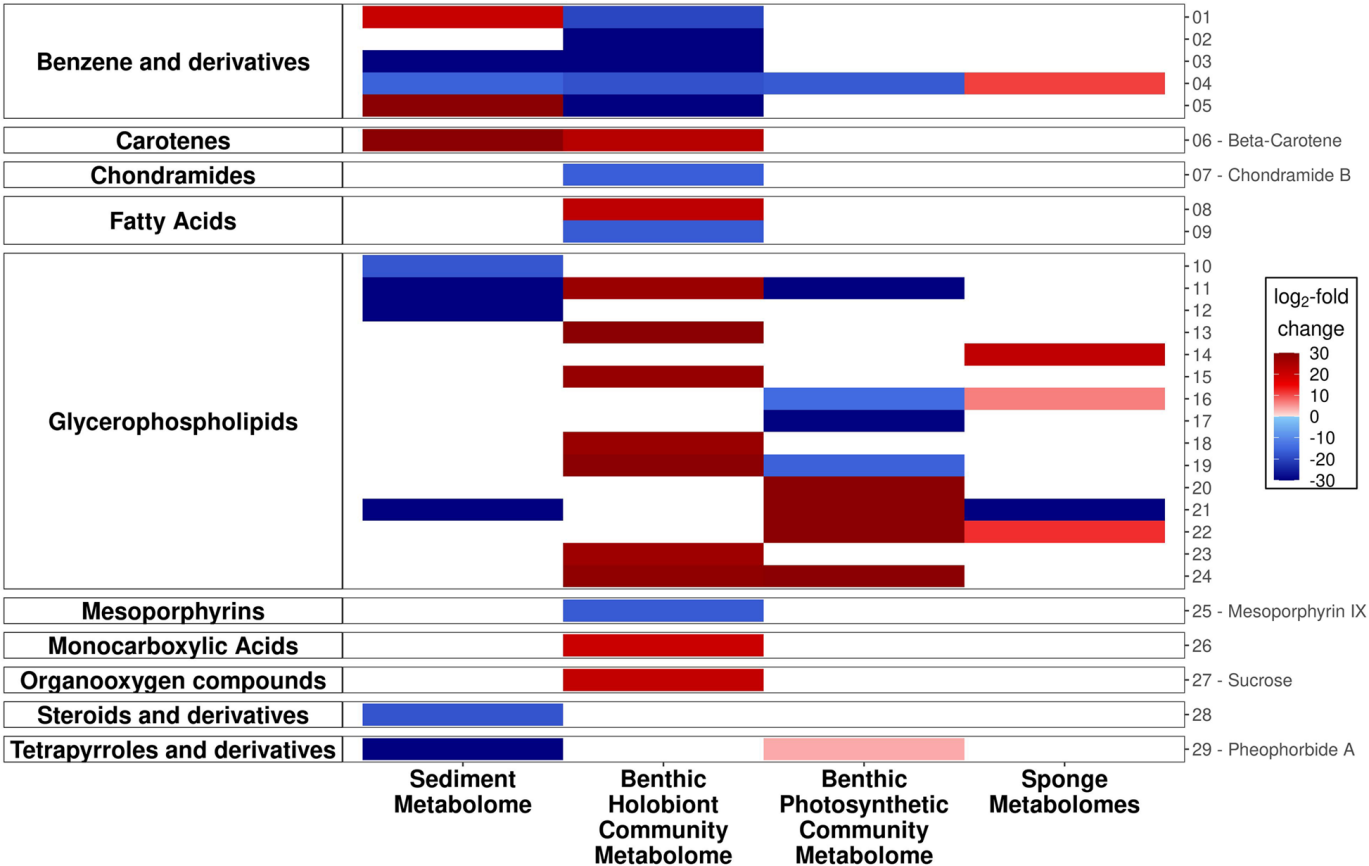
Heat map of significant differential abundance of metabolites with decreasing pH (community and sponge). Identities of metabolites 1–29 are outlined below: 01 — (1S,2S)-2-(methylamino)-1-phenylpropan-1-ol hydrochloride; 02 — benzalkonium chloride (C12); 03 — diphenhydramine|2-benzhydryloxy-N,N-dimethylethanamine; 04 — N,N-diethyl-3-methylbenzamide; 05 — niranthin; 06 — beta-carotene; 07 — chondramide B; 08 — 15(S)-hydroxy-(5Z,8Z,11Z,13E)-eicosatetraenoic acid; 09 — 17(18)-EpETE; 10 — 1-octadecyl-sn-glycero-3-phosphocholine; 11 — 1-palmitoylphosphatidylcholine; 12 — 1-(1Z-hexadecenyl)-sn-glycero-3-phosphocholine; 13 — 1-(9Z-Octadecenoyl)-sn-glycero-3-phosphocholine; 14 — 1-arachidoyl-2-hydroxy-sn-glycero-3-phosphocholine; 15 — 1-hexadecanoyl-sn-glycero-3-phosphocholine; 16 — 1-O-hexadecyl-2-O-(2E-butenoyl)-sn-glyceryl-3-phosphocholine; 17 — 1-octadecyl-sn-glycero-3-phosphocholine; 18 — 1-stearoyl-2-hydroxy-sn-glycero-3-phosphocholine; 19 — lysophosphatidylcholine (LPC 16:0); 20 — lysophosphatidylcholine (LPC 18:1); 21 — lysophosphatidylcholine (LPC 18:2); 22 — lysophosphatidylcholine (LPC 18:3); 23 — lysophosphatidylcholine (LPC 20:5); 24 — lysophosphatidylcholine (LPC 22:6); 25 — mesoporphyrin IX; 26 — 3-indoleacrylic acid; 27 — sucrose; 28 — (R)-4-((3R,5R,8R,9S,10S,12S,13R,14S,17R)-3,12-dihydroxy-10,13-dimethylhexadecahydro-1H-cyclopenta[a]phenanthren-17-yl)pent-2-enoic acid; 29 — pheophorbide A

Fifty-five metabolome libraries were generated from the sediment metabolome, the benthic holobiont community, and the benthic photosynthetic community metabolomes (*n* = 18, 18, 19, respectively). There was no significant compositional difference in these community metabolomes at lower pH (Table S2; Figure S2). There was also no significant effect of pH on compound richness for any community metabolome (Table S7; Figure S3). However, Shannon diversity was significantly lower at lower pH in the benthic holobiont community metabolome (95% *CI* [− 0.07, − 1.18], *p* < 0.05) and significantly higher at lower pH in the sediment metabolome (95% *CI* [0.02, 0.80], *p* < 0.05; Table S8; Figure S3). Decreased pH was associated with significant differences in the abundance of several identified compounds (note that more than 95% of compounds were not identifiable). In the sediment metabolome, glycerophospholipids (lysophosphatidylcholines (LPCs) and phosphocholines) and pheophorbide A (a chlorophyll-derived compound) were less abundant, and beta-carotene was more abundant. In the benthic holobiont community benzene derivatives, chondramide B and mesoporphyrin IX (the latter two both anticarcinogens) were less abundant; a range of glycerophospholipids (again LPCs and phosphocholines), sucrose, and beta-carotene were more abundant. In the benthic photosynthetic community, pheophorbide A was more abundant, and various glycerophospholipids had significantly different abundances, with LPCs mostly having higher abundances and phosphocholines lower abundances (Fig. 4).

Fifty-two metabolome libraries were generated from the two sponge species, with 18 from *Halisarca* sp. and 34 from *Tethya* sp. There was no significant compositional difference in the metabolomes of either sponge at different pH (Table S2; Figure S2). There was also no significant effect of pH on compound richness or Shannon diversity for the sponge metabolomes (Figure S3). Among identified compounds (note that more than 95% of compounds were not identifiable), decreased pH was associated with significantly different abundances in a variety of glycerophospholipids and a significantly increased abundance of a benzene derivate across both sponge species (Fig. 4).

### Holobiont microbial and chemical distinctness

**Fig. 5:**
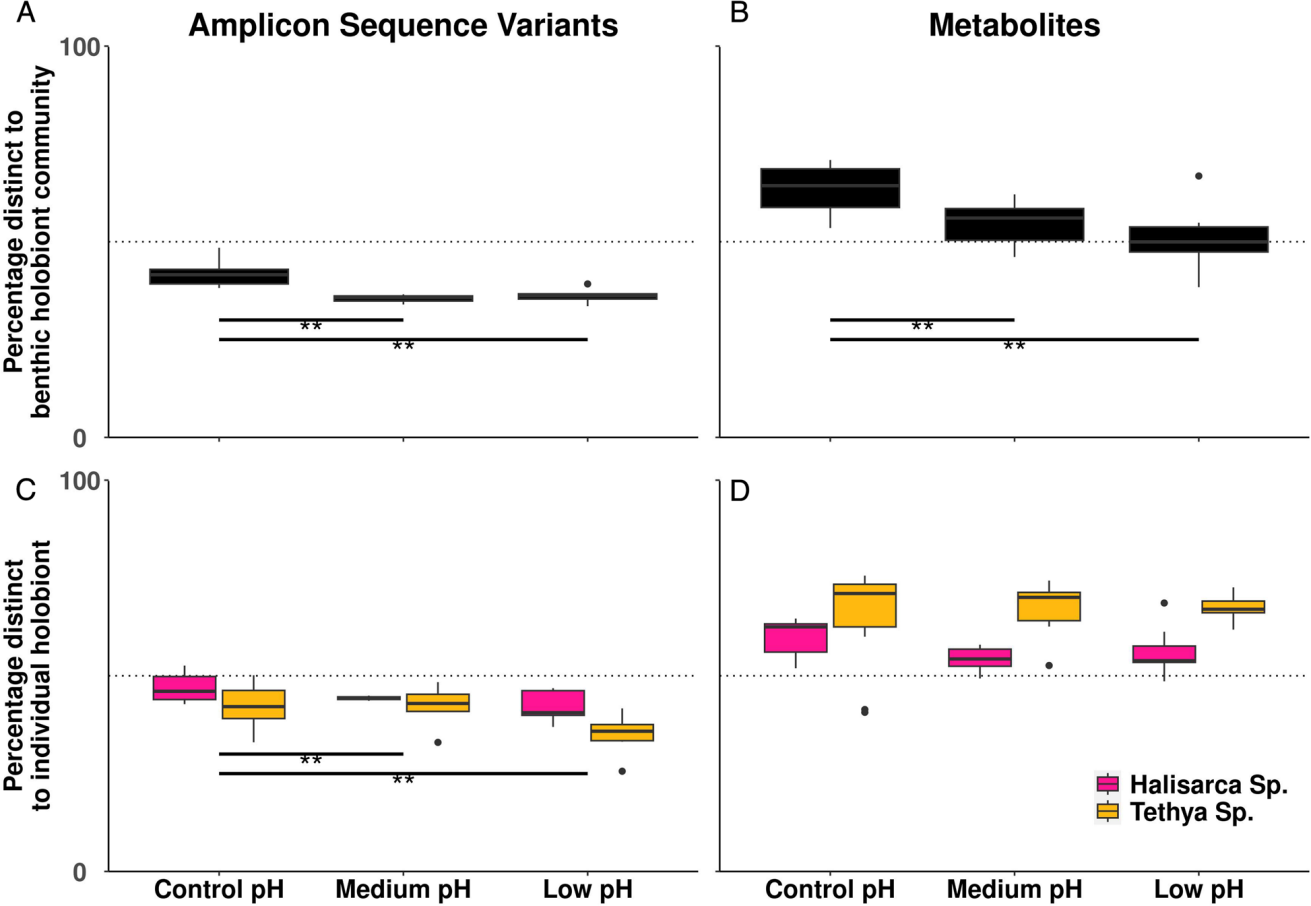
Microbiome and metabolome distinctness of benthic holobiont communities as a function of pH. Microbiome (**A**) and metabolome (**B**) of the benthic holobiont community versus the sediment microbiome and microbiome (**C**) and metabolome (**D**) of the two sponge microbiomes versus the sediment microbiome. Distinctness was calculated as percentage of ASVs/metabolites not shared between microbiomes/metabolomes. Horizontal dotted line indicates 50% distinct. **p-value < 0.01

The proportion of benthic holobiont community microbiome ASVs not found in the sediment microbiome (i.e. holobiont community microbiome distinctness) was lower at lower pH (95% *CI* [− 6.36, − 1.74], *p* < 0.05). The same pattern was observed in the metabolome: the proportion of benthic holobiont community metabolites not found in the sediment (i.e. holobiont community metabolome distinctness) was lower at lower pH (95% *CI* [− 14.46, − 2.83], *p* < 0.05; Fig. 5).

The dispersal probability from the sediment microbiome into the benthic holobiont community microbiome, as measured by the MSNCM (menv weighted by the mixing parameter), was also higher at lower pH. The frequency of occurrence of ASVs across the benthic holobiont community microbiome samples was well described by their abundance in the benthic holobiont community and sediment metacommunities through the MSNCM. However, the fit of the model was poorer at medium and low pH (control: 0.64, medium: 0.29, low: 0.38). The ratio between mholo and menv (both weighted by the mixing parameter) was also lower at lower pH, reflecting an overall increased contribution of sediment microbial abundance to determining the microbiome composition of benthic holobiont communities under OA (Table 2).

**Table 2:** Mixture Sloan neutral community models. Summary of fitted parameters at each pH regime and model fit statisticsMixture weighted

	Mixture-weighted mholo: menv ratio	Mixture-weighted menv	Mixture-weighted mholo	R2
Control pH	1.02	0.1	0.1	0.64
Medium pH	0.59	0.49	0.29	0.29
Low pH	0.26	0.31	0.08	0.38

Change in microbial and chemical distinctness of individual sponge holobionts (*Tethya* sp. and *Halisarca* sp.) with ocean acidification was also analysed to examine whether community-level patterns were observed in individual holobionts. Individual sponge holobiont microbiome distinctness from sediment was lower at lower pH (95% *CI* [− 7.87, − 1.75], *p* < 0.05; Fig. 5). This individual holobiont microbiome distinctness model explained 61% of variation (*R*^2^) compared to 9% of variation (*R*^2^) explained by the equivalent community-level microbiome distinctness model described above. There was no significant effect of pH on sponge metabolome distinctness.

## Discussion

Our results are consistent with the simplification of the algal community seen under OA elsewhere [73]. Our results further demonstrate that this simplification effect extends across the entire benthic photosynthetic community holobiome as algal-associated microbes also decline in Shannon diversity with OA. While we do not observe an overall compositional shift in the benthic photosynthetic community metabolome, implying that photosynthetic function is largely conserved through these changes, we do see shifts in specific metabolites. Increasing concentration of chlorophyll-derived pheophorbide A suggests increased chlorophyll turnover [74], consistent with the doubling of macroalgal benthic cover previously observed under OA at these sites [18]. We also see significant increases in sucrose in the benthic holobiont community under OA, potentially related to sugar-enriched dissolved organic carbon (DOC) released by these algae.

Both findings are consistent with the initial steps in the DDAM mechanism of microbialisation [26], whereby increased DOC is released from fleshy algae, in this case *Sargassum* [19]. We did not, however, test for the increased microbial biomass previously shown to result from increased DOC stimulating the microbial loop and causing a shift in ecosystem trophic structure [26, 75]. The main microbial phyla that we observed to increase in abundance in our community samples (i.e. not sponge samples) with low pH (Desulfobacterota and *WOR-3*) differ from those observed by Haas [26] (Alphaproteobacteria, Gammaproteobacteria, and Bacteroidetes). However, this difference in specific taxonomic patterns in response to different stressors, across different sites, and when sampling different substrates (water vs. sediment) is expected and is a key motivation for seeking to identify more general ecosystem-level effects of stressors, such as microbiome distinctness.

The data presented here provide the first evidence of declining holobiont community distinctness in microbes and metabolites under OA. Our results build on the evidence that OA changes holobiont microbiomes [76] by demonstrating a systematic decline of a distinct benthic holobiont community microbiome, such that it becomes more compositionally similar to the sediment microbiome. The composition of the sediment microbiome in our study is representative of surface sediments from the region [77] and global analyses of marine sediments [78]. Specifically, the results of the MSNCM suggest that this effect may result from the benthic holobiont community being increasingly colonised by sediment microbes as pH decreases. A similar effect has previously been observed for specific sponge [79] and coral [80, 81] holobionts in response to different environmental stressors, but has not previously been observed at the community level. While organism-level studies provide information about the response of key species (e.g. habitat builders) to environmental change, a holistic approach is needed to accurately evaluate and predict impacts on coral reefs. The synthesis of knowledge across scales, from individual microbes and holobionts to ecosystem-wide communities and processes, has recently been called for by multiple authors [29, 75, 82]. Autonomous reef monitoring structures (ARMS) provide a novel tool for taking this “nested ecosystem approach” and conducting in situ experiments.

We explain the decline in the distinctness of the benthic holobiont community from the sediment microbiome as being caused by increased opportunities for colonisation of benthic holobiont communities by environmental microbes due to microbialisation. However, individual macrobes under stress may also become less able to regulate their microbiomes, while colonisation opportunities remain constant. In extreme cases, this inability to regulate the microbiome can result in traumatic dysbiosis [80], a more heterogeneous microbiome (Anna Karenina principle, [83], and host death. However, it is unlikely that this process is the major contributor to the observed community-level effect seen here because macrobe community compositions have already shifted under OA conditions at these vent sites, with an increased dominance of taxa that are less impacted by the stressor [18, 84]. Therefore, the notion that the majority of the macrobe community is experiencing dysbiosis associated with acute organism-level stress seems unlikely. For example, some sponges are known to thrive under OA, and do not exhibit evidence of organismal stress [85–87]. In this study, the two sponge holobionts individually analysed (*Tethya* sp. and *Halisarca* sp.) showed reduced distinctness of their microbiomes from the sediment microbiome under OA. However, neither showed evidence of increased compositional heterogeneity of their microbiomes as expected under dysbiosis by the Anna Karenina principle, which predicts organism-level stress to reduce the ability of macrobes to regulate their microbiomes [83]. In addition, metabolomes for these two sponges do not become significantly less distinct from the sediment under OA, which is consistent with the hypothesis that these sponges were not under stress.

Colonisation of holobionts by environmental microbes may support the resilience of macrobe communities, as it has been shown to allow some hosts to acclimatise to new environmental conditions, for example by allowing the host to make use of changing energy sources, and facilitate greater adaptation than can be afforded by host phenotypic plasticity [88, 89]. Different degrees of microbial restructuring observed among different sponge species indicate that horizontal transmission differs between species, and these variations affect the ability of sponges to persist under OA conditions [12, 90]. Here, we find significant increases in Cyanobacteria associated with sponges under OA conditions, which can contribute > 50% of a sponge’s carbon demand [91] and likely provide at least some sponge species with enhanced scope for growth in these seep environments [12]. We also find significant increases in *Desulfobacterota* in the benthic photosynthetic community, benthic holobiont community, and sediment; this phylum includes many organisms capable of reducing sulphur compounds [92, 93]. Only one of the two ocean vents (Dobu) is known to release hydrogen sulphide [18], so this does not explain the increase in Desulfobacterota observed across sites. We note that while we do not identify changes in the differential abundance of any metabolites with known roles in sulphur cycling, this is not evidence of their absence due to the low level (less than 5%) of identification of metabolites. While their role here is unknown, coral reefs are important hotspots of marine sulphur and increased sulphate reduction rates of marine microbial communities have been found to occur between a pH of 6 and 7 [94], suggesting that rates may increase with OA. As the marine sulphur cycle is a quintessential example of algal–bacterial interactions [95], it will be important for future studies to investigate the impact of algal-derived microbialisation on the marine sulphur cycle, especially as new components and pathways in the sulphur cycle are still being identified [96].

One would expect the dynamics of microbes and holobionts to be universal to all ecosystems [36, 97, 98], though they may emerge from different organism-level interactions. Therefore, microbialisation, and the observable property of declining holobiont community distinctness under environmental change, could represent a universal ecosystem stress response. Identifying such a general, undesirable response (microbialised ecosystems typically have lower intrinsic and use values [99]) and a metric of ecosystem change has clear benefits to policy and evaluation. For example, ecosystem change and the associated risk of ecosystem collapse are the underpinning concept leveraged for the IUCN Red List of Ecosystems [100], but defining collapse for each ecosystem individually is a time-consuming and contentiously value-laden task [101, 102]. Furthermore, as microbial communities respond rapidly to environmental change, microbial bioindicators could provide signatures of change with the speed and resolution to allow real-time responses by ecosystem managers. Generating predictions of ecosystem change based on a mechanistic understanding of all organism-level effects of stressors remains unrealistic [17, 103]. Therefore, identifying general ecosystem-level changes under stress presents a promising route towards a more efficient predictive ecosystem science, responding to the urgent needs of the biodiversity and climate crisis.

### Supplementary Information

**Additional file 1:** **Table S1.** Number of sequences retained at each step of denoising (implemented in DADA2); samples with fewer than 50,000 denoised sequences (ASVs) and duplicate samples were removed prior to analysis. **Table S2. **Effect size and significance of factors in PERMANOVAs describing the compositional differences among sites, and along the pH gradient for all multiomic data types; site fit as a blocking factor, except in the case of Halisarca sp. sponge fractions, which were only collected at one site. A *p*-value less than 0.05 indicates that the factor significantly affects composition. Bonferroni correction was applied to all PERMANOVA. **Table S3. **ASV estimated richness betta mixed model: fixed effects listed, random effect of locality. Model Explanatory Power: test statistic = 117.2, *p*<0.05. A *p*-value less than 0.05 indicates that the explanatory variable significantly affects ASV richness. All values in the table are reported to two significant figures. **Table S4. **Phylum Shannon diversity betta mixed model: fixed effects listed, random effect of locality. Model Explanatory Power: test statistic = 3197.94, *p*<0.05. A p-value less than 0.05 indicates that the explanatory variable significantly affects metabolite richness. All values in the table are reported to two significant figures. **Table S5. **ASV Shannon diversity betta mixed model: fixed effects listed, random effect of locality. R Squared (conditional)= 88.8%. A *p*-value less than 0.05 indicates that the explanatory variable significantly affects metabolite richness. All values in the table are reported to two significant figures. **Table S6. **Summary of tests and results for 16S rRNA gene and metabolomic data. **Table S7. **Compound richness linear mixed model: fixed effects listed, random effect of locality. R Squared (conditional)= 24.5%. A *p*-value less than 0.05 indicates that the explanatory variable significantly affects ASV richness. All values in the table are reported to two significant figures. **Table S8. **Compound Shannon diversity linear mixed model: fixed effects listed, random effect of locality. R Squared (conditional)= 32.0%. A *p*-value less than 0.05 indicates that the explanatory variable significantly affects ASV richness. All values in the table are reported to two significant figures. **Fig. S1.** Visual representation of read abundance from the ARMS 23S rRNA gene and 16S rRNA gene metabarcoding dataset. Data are aggregated across biological replicates to present the average composition of each fraction, at each pH, showing phylum (in white text) and class (in grey text). **Fig. S2.** NMDS of microbiome and metabolome composition of all fractions across the pH gradient, calculated using Morisita dissimilarity. **Fig. S3.** Microbial and chemical richness and Shannon diversity boxplots for all fractions across the pH gradient.

## Data Availability

The metabarcoding datasets are available from the NCBI Sequence Read Archive repository. 16S rRNA gene data is available under BioProject ID PRJNA945340 (http://www.ncbi.nlm.nih.gov/bioproject/945340) and 23S rRNA gene data is available under BioProject ID PRJNA945259 (http://www.ncbi.nlm.nih.gov/bioproject/945259). The mass spectrometry data is available at the GNPS MassIVE repository under MassIVE ID: MSV000080572 (https://gnps.ucsd.edu/ProteoSAFe/status.jsp?task=f5c6591769d541a68fdb8bb201532054). R scripts used in the bioinformatic pipeline are archived at https://doi.org/10.5281/zenodo.7740559 and available at https://github.com/J-Cos/BioinformaticPipeline. R scripts for the statistical analysis are archived at https://doi.org/10.5281/zenodo.8280507 and available at: https://github.com/J-Cos/Paper_PNG.

## References

[1] Landschützer P, et al. Recent variability of the global ocean carbon sink. Glob Biogeochem Cycl. 2014. 10.1002/2014GB004853.

[2] Gaylord B, et al. Ocean acidification through the lens of ecological theory. Ecology. 2015. 10.1890/14-0802.1. 10.1890/14-0802.1 26236884

[3] Doney SC, et al. The impacts of ocean acidification on marine ecosystems and reliant human communities. Annu Rev Environ Resourc. 2020. 10.1146/annurev-environ-012320-083019.

[4] Hughes TP, et al. Coral reefs in the Anthropocene. Nature. 2017. 10.1038/nature22901. 10.1038/nature22901 28569801

[5] Bourne DG, Webster NS. Coral reef bacterial communities. Prokaryotes–Prokaryotic Communities Ecophysiol. 2013. 10.1007/978-3-642-30123-0_48.

[6] Lesser MP, Blakemore RP (1990). Description of a novel symbiotic bacterium from the brittle star Amphipholis squamata. Appl Environ Microbiol.

[7] Rosenberg E (2007). The role of microorganisms in coral health, disease and evolution. Nature Rev Microbiol.

[8] Roeselers G, Newton IL (2012). On the evolutionary ecology of symbioses between chemosynthetic bacteria and bivalves. Appl Microbiol Biotechnol.

[9] Schmitt S (2012). Assessing the complex sponge microbiota: core, variable and species-specific bacterial communities in marine sponges. ISME J.

[10] Hoadley KD (2015). Physiological response to elevated temperature and *p*CO_2_ varies across four Pacific coral species: understanding the unique host+symbiont response. Sci Rep.

[11] Webster NS, Reusch TBH. Microbial contributions to the persistence of coral reefs. ISME J. 2017. 10.1038/ismej.2017.66. 10.1038/ismej.2017.66 PMC5607359 28509908

[12] Morrow KM, et al. Natural volcanic CO_2_ seeps reveal future trajectories for host-microbial associations in corals and sponges. ISME J. 2015. 10.1038/ismej.2014.188. 10.1038/ismej.2014.188 PMC4817704 25325380

[13] Rädecker N, et al. Nitrogen cycling in corals: the key to understanding holobiont functioning? Trends Microbiol. 2015. 10.1016/j.tim.2015.03.008. 10.1016/j.tim.2015.03.008 25868684

[14] Hu C, et al. Effect of ocean acidification on bacterial metabolic activity and community composition in oligotrophic oceans, inferred from short-term bioassays. Front Microbiol. 2021. 10.3389/fmicb.2021.583982. 10.3389/fmicb.2021.583982 PMC7952631 33716995

[15] Burkepile DE, Thurber RV. The long arm of species loss: how will defaunation disrupt ecosystems down to the microbial scale? Bioscience. 2019. 10.1093/biosci/biz047.

[16] Cavicchioli R, et al. Scientists’ warning to humanity: microorganisms and climate change. Nature Rev Microbiol. 2019. 10.1038/s41579-019-0222-5. 10.1038/s41579-019-0222-5 PMC7136171 31213707

[17] Simmons BI, et al. Refocusing multiple stressor research around the targets and scales of ecological impacts. Nat Ecol Evol. 2021. 10.1038/s41559-021-01547-4. 10.1038/s41559-021-01547-4 34556829

[18] Fabricius KE, et al. Losers and winners in coral reefs acclimatized to elevated carbon dioxide concentrations. Nat Clim Change. 2011. 10.1038/nclimate1122.

[19] Diaz-Pulido G, Barrón C. CO2 enrichment stimulates dissolved organic carbon release in coral reef macroalgae. J Phycol. 2020. 10.1111/jpy.13002. 10.1111/jpy.13002 32279320

[20] Saint-Béat B, et al. Trophic networks: how do theories link ecosystem structure and functioning to stability properties? A review. Ecol Indicat. 2015. 10.1016/j.ecolind.2014.12.017.

[21] Bierwagen SL, et al. Trophodynamics as a tool for understanding coral reef ecosystems. Front Mar Sci. 2018. 10.3389/fmars.2018.00024.

[22] Vizzini S, et al. Ocean acidification as a driver of community simplification via the collapse of higher-order and rise of lower-order consumers. Sci Rep. 2017. 10.1038/s41598-017-03802-w. 10.1038/s41598-017-03802-w PMC5481442 28642608

[23] McDole T, et al. Assessing coral reefs on a Pacific-wide scale using the microbialization score. PLoS One. 2012 10.1371/journal.pone.0043233. 10.1371/journal.pone.0043233 PMC3436891 22970122

[24] Hill TS, Hoogenboom MO (2022). The indirect effects of ocean acidification on corals and coral communities. Coral Reefs.

[25] Jackson JBC, et al. Historical overfishing and the recent collapse of coastal ecosystems. Science. 2001. 10.1126/science.1059199. 10.1126/science.1059199 11474098

[26] Haas AF, et al. Global microbialization of coral reefs. Nat Microbiol. 2016. 10.1038/nmicrobiol.2016.42. 10.1038/nmicrobiol.2016.42 27572833

[27] Yao L, et al. Global microbial carbonate proliferation after the end-Devonian mass extinction: mainly controlled by demise of skeletal bioconstructors. Sci Rep. 2016. 10.1038/srep39694. 10.1038/srep39694 PMC5180103 28009013

[28] Butterfield NJ. Animals and the invention of the phanerozoic Earth system. Trends Ecol Evol. 2011. 10.1016/j.tree.2010.11.012. 10.1016/j.tree.2010.11.012 21190752

[29] Vanwonterghem I, Webster NS. Coral reef microorganisms in a changing climate. iScience. 2020. 10.1016/j.isci.2020.100972. 10.1016/j.isci.2020.100972 PMC7096749 32208346

[30] Hall-Spencer JM, et al. Volcanic carbon dioxide vents show ecosystem effects of ocean acidification. Nature. 2008. 10.1038/nature07051. 10.1038/nature07051 18536730

[31] Rubal M, et al. Mollusc diversity associated with the non-indigenous macroalga *Asparagopsis armata* Harvey, 1855 along the Atlantic Coast of the Iberian Peninsula. Mar Environ Res. 2018. 10.1016/j.marenvres.2018.02.025. 10.1016/j.marenvres.2018.02.025 29496205

[32] Rix L, et al. Reef sponges facilitate the transfer of coral-derived organic matter to their associated fauna via the sponge loop. Mar Ecol Prog Ser. 2018. 10.3354/meps12443.

[33] Steffan SA, Dharampal PS. Undead food-webs: integrating microbes into the food-chain. Food Webs. 2019. 10.1016/j.fooweb.2018.e00111.

[34] Herrmann NC, Stroud JT, Losos JB. The evolution of “ecological release” into the 21st century. Trends Ecol Evol. 2021. 10.1016/j.tree.2020.10.019. 10.1016/j.tree.2020.10.019 33223276

[35] Bosch TCG, Miller DJ (2016). The holobiont imperative: perspectives from early emerging animals.

[36] Simon JC, et al. Host-microbiota interactions: from holobiont theory to analysis. Microbiome. 2019. 10.1186/s40168-019-0619-4. 10.1186/s40168-019-0619-4 PMC6330386 30635058

[37] Singh BK, Liu H, Trivedi P. Eco-holobiont: a new concept to identify drivers of host-associated microorganisms. Environ Microbiol. 2020. 10.1111/1462-2920.14900. 10.1111/1462-2920.14900 31849163

[38] Zaneveld JR, et al. Overfishing and nutrient pollution interact with temperature to disrupt coral reefs down to microbial scales. Nature Comm. 2016. 10.1038/ncomms11833. 10.1038/ncomms11833 PMC4899628 27270557

[39] Burkepile DE, et al. Chemically mediated competition between microbes and animals: microbes as consumers in food webs. Ecology. 2006. 10.1890/0012-9658(2006)87[2821:CMCBMA]2.0.CO;2. 10.1890/0012-9658(2006)87[2821:cmcbma]2.0.co;2 17168026

[40] Longford SR, et al. Interactions within the microbiome alter microbial interactions with host chemical defences and affect disease in a marine holobiont. Sci Rep. 2019. 10.1038/s41598-018-37062-z. 10.1038/s41598-018-37062-z PMC6361982 30718608

[41] Pearman JK, et al. Disentangling the complex microbial community of coral reefs using standardized autonomous reef monitoring structures (ARMS). Molec Ecol. 2019. 10.1111/mec.15167. 10.1111/mec.15167 PMC6851789 31281998

[42] Ip YCA, et al. ‘Seq’ and ARMS shall find: DNA (meta)barcoding of autonomous reef monitoring structures across the tree of life uncovers hidden cryptobiome of tropical urban coral reefs. Molec Ecol. 2022. 10.1111/mec.16568. 10.1111/mec.16568 35716352

[43] Steyaert M, et al. Remote reef cryptobenthic diversity: integrating autonomous reef monitoring structures and in situ environmental parameters. Front Mar Sci. 2022. 10.3389/fmars.2022.932375.

[44] Ransome E, et al. The importance of standardization for biodiversity comparisons: a case study using autonomous reef monitoring structures (ARMS) and metabarcoding to measure cryptic diversity on Mo’orea coral reefs. French Polynesia PLoS One. 2017. 10.1371/journal.pone.0175066. 10.1371/journal.pone.0175066 PMC5400227 28430780

[45] Foo SA, Byrne M. Forecasting impacts of ocean acidification on marine communities: utilizing volcanic CO_2_ vents as natural laboratories. Glob Change Biol. 2021. 10.1111/gcb.15528. 10.1111/gcb.15528 33501734

[46] Makiola A, et al. Key questions for next-generation biomonitoring. Front Environ Sci. 2020. 10.3389/fenvs.2019.00197.

[47] Hartmann AC, et al. Meta-mass shift chemical profiling of metabolomes from coral reefs. Proc Nat Acad Sci. 2017. 10.1073/pnas.1710248114. 10.1073/pnas.1710248114 PMC5676912 29078340

[48] Plaisance L, et al. Effects of low pH on the coral reef cryptic invertebrate communities near CO_2_ vents in Papua New Guinea. PLoS ONE. 2021. 10.1371/journal.pone.0258725. 10.1371/journal.pone.0258725 PMC8673656 34910721

[49] Gloeckner V, et al. The HMA-LMA dichotomy revisited: an electron microscopical survey of 56 sponge species. Biol Bull. 2014. 10.1086/BBLv227n1p78. 10.1086/BBLv227n1p78 25216505

[50] Lesser MP, et al. Depth-dependent detritus production in the sponge. Halisarca caerulea Limnol Oceanogr. 2020. 10.1002/lno.11384.

[51] Caporaso JG (2012). Ultra-high-throughput microbial community analysis on the Illumina HiSeq and MiSeq platforms. ISME J.

[52] Marcelino VR, Verbruggen H. Multi-marker metabarcoding of coral skeletons reveals a rich microbiome and diverse evolutionary origins of endolithic algae. Sci Rep. 2016. 10.1038/srep31508. 10.1038/srep31508 PMC4992875 27545322

[53] Quinn RA, et al. Metabolomics of reef benthic interactions reveals a bioactive lipid involved in coral defence. Proc Roy Soc B Biol Sci. 2016. 10.1098/rspb.2016.0469. 10.1098/rspb.2016.0469 PMC4855392 27122568

[54] Callahan BJ, et al. DADA2: high-resolution sample inference from Illumina amplicon data. Nat Meth. 2016. 10.1038/nmeth.3869. 10.1038/nmeth.3869 PMC4927377 27214047

[55] Brandt MI, et al. Bioinformatic pipelines combining denoising and clustering tools allow for more comprehensive prokaryotic and eukaryotic metabarcoding. Molec Ecol Resour. 2021. 10.1111/1755-0998.13398. 10.1111/1755-0998.13398 33835712

[56] Murali A, Bhargava A, Wright ES. IDTAXA: a novel approach for accurate taxonomic classification of microbiome sequences. Microbiome. 2018. 10.1186/s40168-018-0521-5. 10.1186/s40168-018-0521-5 PMC6085705 30092815

[57] Parks DH, et al. GTDB: an ongoing census of bacterial and archaeal diversity through a phylogenetically consistent, rank normalized and complete genome-based taxonomy. Nucl Acids Res. 2022. 10.1093/nar/gkab776. 10.1093/nar/gkab776 PMC8728215 34520557

[58] Djemiel C, et al. µgreen-db: a reference database for the 23S rRNA gene of eukaryotic plastids and cyanobacteria. Sci Rep. 2020. 10.1038/s41598-020-62555-1. 10.1038/s41598-020-62555-1 PMC7125122 32246067

[59] McMurdie PJ, Holmes S. Waste not, want not: why rarefying microbiome data is inadmissible. PLoS Comput Biol. 2014. 10.1371/journal.pcbi.1003531. 10.1371/journal.pcbi.1003531 PMC3974642 24699258

[60] Beck J, Holloway JD, Schwanghart W. Undersampling and the measurement of beta diversity. Meth Ecol Evol. 2013. 10.1111/2041-210x.12023.

[61] Willis A, Bunge J, Whitman T. Improved detection of changes in species richness in high diversity microbial communities. J Roy Stat Soc Ser C Appl Stat. 2017. 10.1111/rssc.12206.

[62] Willis AD, Martin BD. Estimating diversity in networked ecological communities. Biostatistics. 2022. 10.1093/biostatistics/kxaa015. 10.1093/biostatistics/kxaa015 PMC8759443 32432696

[63] Love MI, Huber W, Anders S. Moderated estimation of fold change and dispersion for RNA-seq data with DESeq2. Genome Biol. 2014. 10.1186/s13059-014-0550-8. 10.1186/s13059-014-0550-8 PMC4302049 25516281

[64] Sloan WT, et al. Quantifying the roles of immigration and chance in shaping prokaryote community structure. Environ Microbiol. 2006. 10.1111/j.1462-2920.2005.00956.x. 10.1111/j.1462-2920.2005.00956.x 16584484

[65] Burns AR, et al. Contribution of neutral processes to the assembly of gut microbial communities in the zebrafish over host development. ISME J. 2016. 10.1038/ismej.2015.142. 10.1038/ismej.2015.142 PMC4817674 26296066

[66] R Core Team. R: a language and environment for statistical computing. Vienna: R Foundation for Statistical Computing; 2022. Available from: http://www.R-project.org.

[67] McMurdie PJ, Holmes S. phyloseq: an R package for reproducible interactive analysis and graphics of microbiome census data. PLoS One. 2013. 10.1371/journal.pone.0061217. 10.1371/journal.pone.0061217 PMC3632530 23630581

[68] Oksanen J, et al. vegan: community ecology package. R package version 2.6–4. 2022. Available from: https://CRAN.R-project.org/package=vegan.

[69] Bates D (2015). Fitting linear mixed-effects models using lme4. J Stat Software.

[70] Bartoń K. MuMIn: multi-model inference. R package version 1.47.1. 2022. Available from: https://CRAN.R-project.org/package=MuMIn.

[71] Elzhov TV, et al. minpack.lm: R interface to the Levenberg-Marquardt nonlinear least-squares algorithm found in MINPACK, plus support for bounds. R package version 1.2–2. 2022. Available from: https://CRAN.R-project.org/package=minpack.lm.

[72] Harrell JF. Hmisc: Harrell miscellaneous. R package version 4.7–1. 2022. Available from: https://CRAN.R-project.org/package=Hmisc.

[73] Harvey BP, et al. Ocean acidification locks algal communities in a species-poor early successional stage. Glob Change Biol. 2021. 10.1111/gcb.15455. 10.1111/gcb.15455 33423359

[74] Lauritano C, et al. Lysophosphatidylcholines and chlorophyll-derived molecules from the diatom *Cylindrotheca closterium* with anti-inflammatory activity. Mar Drugs. 2020. 10.3390/md18030166. 10.3390/md18030166 PMC7143213 32192075

[75] Wegley Kelly L, et al. Molecular commerce on coral reefs: using metabolomics to reveal biochemical exchanges underlying holobiont biology and the ecology of coastal ecosystems. Front Mar Sci. 2021. 10.3389/fmars.2021.630799.

[76] Rastelli E, et al. A high biodiversity mitigates the impact of ocean acidification on hard-bottom ecosystems. Sci Rep. 2020. 10.1038/s41598-020-59886-4. 10.1038/s41598-020-59886-4 PMC7031329 32076065

[77] Raulf FF, et al. Changes in microbial communities in coastal sediments along natural CO_2_ gradients at a volcanic vent in Papua New Guinea. Environ Microbiol. 2015. 10.1111/1462-2920.12729. 10.1111/1462-2920.12729 25471738

[78] Hoshino T, et al. Global diversity of microbial communities in marine sediment. Proc Nat Acad Sci. 2020. 10.1073/pnas.1919139117. 10.1073/pnas.1919139117 PMC7959581 33077589

[79] Pita L, et al. The sponge holobiont in a changing ocean: from microbes to ecosystems. Microbiome. 2018. 10.1186/s40168-018-0428-1. 10.1186/s40168-018-0428-1 PMC5845141 29523192

[80] Boilard A, et al. Defining coral bleaching as a microbial dysbiosis within the coral holobiont. Microorganisms. 2020. 10.3390/microorganisms8111682. 10.3390/microorganisms8111682 PMC7692791 33138319

[81] MacKnight NJ, et al. Microbial dysbiosis reflects disease resistance in diverse coral species. Comm Biol. 2021. 10.1038/s42003-021-02163-5. 10.1038/s42003-021-02163-5 PMC8175568 34083722

[82] Garren M, Azam F. New directions in coral reef microbial ecology. Environ Microbiol. 2012. 10.1111/j.1462-2920.2011.02597.x. 10.1111/j.1462-2920.2011.02597.x 21955796

[83] Zaneveld JR, McMinds R, Thurber RV. Stress and stability: applying the Anna Karenina principle to animal microbiomes. Nat Microbiol. 2017. 10.1038/nmicrobiol.2017.121. 10.1038/nmicrobiol.2017.121 28836573

[84] Hempson TN, et al. Ecosystem regime shifts disrupt trophic structure. Ecol Appl. 2018. 10.1002/eap.1639. 10.1002/eap.1639 29035010

[85] Kandler NM, et al. In situ responses of the sponge microbiome to ocean acidification. FEMS Microbiol Ecol. 2018. 10.1093/femsec/fiy205. 10.1093/femsec/fiy205 30304402

[86] Botté ES, et al. Changes in the metabolic potential of the sponge microbiome under ocean acidification. Nat Comm. 2019. 10.1038/s41467-019-12156-y. 10.1038/s41467-019-12156-y PMC6742649 31515490

[87] Page HN, et al. Ocean acidification and direct interactions affect coral, macroalga, and sponge growth in the Florida Keys. J Mar Sci Engin. 2021. 10.3390/jmse9070739.

[88] Bourne D, et al. Changes in coral-associated microbial communities during a bleaching event. ISME J. 2008. 10.1038/ismej.2007.112. 10.1038/ismej.2007.112 18059490

[89] Voolstra CR, Ziegler M. Adapting with microbial help: microbiome flexibility facilitates rapid responses to environmental change. BioEssays. 2020. 10.1002/bies.202000004. 10.1002/bies.202000004 32548850

[90] Ribes M, et al. Restructuring of the sponge microbiome favors tolerance to ocean acidification. Environ Microbiol Rep. 2016. 10.1111/1758-2229.12430. 10.1111/1758-2229.12430 27264698

[91] Freeman CJ, Thacker RW. Complex interactions between marine sponges and their symbiotic microbial communities. Limnol Oceanogr. 2011. 10.4319/lo.2011.56.5.1577.

[92] Waite DW, et al. Proposal to reclassify the proteobacterial classes Deltaproteobacteria and Oligoflexia, and the phylum Thermodesulfobacteria into four phyla reflecting major functional capabilities. Int J Syst Evol Microbiol. 2020. 10.1099/ijsem.0.004213. 10.1099/ijsem.0.004213 33151140

[93] Hahn CR, et al. Microbial diversity and sulfur cycling in an early earth analogue: from ancient novelty to modern commonality. MBio. 2022. 10.1128/mbio.00016-22. 10.1128/mbio.00016-22 PMC9040765 35258328

[94] Bayraktarov E, et al. The pH and pCO_2_ dependence of sulfate reduction in shallow-sea hydrothermal CO_2_-venting sediments (Milos Island, Greece). Front Microbiol. 2013. 10.3389/fmicb.2013.00111. 10.3389/fmicb.2013.00111 PMC3647119 23658555

[95] Cirri E, Pohnert G. Algae−bacteria interactions that balance the planktonic microbiome. New Phytol. 2019. 10.1111/nph.15765. 10.1111/nph.15765 30825329

[96] Thume K, et al. The metabolite dimethylsulfoxonium propionate extends the marine organosulfur cycle. Nature. 2018. 10.1038/s41586-018-0675-0. 10.1038/s41586-018-0675-0 30429546

[97] Falkowski PG, Fenchel T, Delong EF. The microbial engines that drive earth’s biogeochemical cycles. Science. 2008. 10.1126/science.1153213. 10.1126/science.1153213 18497287

[98] Jousset A, et al. Where less may be more: how the rare biosphere pulls ecosystems strings. ISME J. 2017. 10.1038/ismej.2016.174. 10.1038/ismej.2016.174 PMC5364357 28072420

[99] Jackson JBC. Ecological extinction and evolution in the brave new ocean. Proc Nat Acad Sci. 2008. 10.1073/pnas.0802812105. 10.1073/pnas.0802812105 PMC2556419 18695220

[100] Keith DA, et al. Scientific foundations for an IUCN Red List of Ecosystems. PLoS One. 2013. 10.1371/journal.pone.0062111. 10.1371/journal.pone.0062111 PMC3648534 23667454

[101] Boitani L, Mace GM, Rondinini C. Challenging the scientific foundations for an IUCN Red List of Ecosystems. Conserv Lett. 2015. 10.1111/conl.12111.

[102] Glasl B, et al. Microbial indicators of environmental perturbations in coral reef ecosystems. Microbiome. 2019. 10.1186/s40168-019-0705-7. 10.1186/s40168-019-0705-7 PMC6588946 31227022

[103] Harfoot MBJ. et al. Emergent global patterns of ecosystem structure and function from a mechanistic general ecosystem model. PLoS Biol. 2014. 10.1371/journal.pbio.1001841. 10.1371/journal.pbio.1001841 PMC3995663 24756001

