# Supplementary material for: Decline of a distinct coral reef holobiont community under ocean acidification
Source: Microbiome. 2024 Apr 17;12:75. doi: 10.1186/s40168-023-01683-y (PMC11022381; doi:10.1186/s40168-023-01683-y)
Supplement: Supplementary file 2 — Additional file 1: Table S1. Number of sequences retained at each step of denoising (implemented in DADA2); samples with fewer than 50,000 denoised sequences (ASVs) and duplicate samples were removed prior to analysis. Table S2. Effect size and significance of factors in PERMANOVAs describing the compositional differences among sites, and along the pH gradient for all multiomic data types; site fit as a blocking factor, except in the case of Halisarca sp. sponge fractions, which were only collected at one site. A p-value less than 0.05 indicates that the factor significantly affects composition. Bonferroni correction was applied to all PERMANOVA. Table S3. ASV estimated richness betta mixed model: fixed effects listed, random effect of locality. Model Explanatory Power: test statistic = 117.2, p<0.05. A p-value less than 0.05 indicates that the explanatory variable significantly affects ASV richness. All values in the table are reported to two significant figures. Table S4. Phylum Shannon diversity betta mixed model: fixed effects listed, random effect of locality. Model Explanatory Power: test statistic = 3197.94, p<0.05. A p-value less than 0.05 indicates that the explanatory variable significantly affects metabolite richness. All values in the table are reported to two significant figures. Table S5. ASV Shannon diversity betta mixed model: fixed effects listed, random effect of locality. R Squared (conditional)= 88.8%. A p-value less than 0.05 indicates that the explanatory variable significantly affects metabolite richness. All values in the table are reported to two significant figures. Table S6. Summary of tests and results for 16S rRNA gene and metabolomic data. Table S7. Compound richness linear mixed model: fixed effects listed, random effect of locality. R Squared (conditional)= 24.5%. A p-value less than 0.05 indicates that the explanatory variable significantly affects ASV richness. All values in the table are reported to two significant figures. Table S [file 40168_2023_1683_MOESM1_ESM.docx]

Supplementary Material for “Decline of a distinct coral reef holobiont community under ocean acidification”

## **Supplementary Tables**

**Table S1:** Number of sequences retained at each step of denoising (implemented in DADA2); samples with fewer than 50,000 denoised sequences (ASVs) and duplicate samples were removed prior to analysis.

**16S gene rRNA Samples**

|  | input | filtered | denoisedF | denoisedR | merged | non-chimeric |
| --- | --- | --- | --- | --- | --- | --- |
| Algae_Con_Dobu_103_26_L001 | 253403 | 245650 | 242101 | 241621 | 207634 | 186504 |
| Algae_Con_Dobu_104_40_L001 | 259020 | 252481 | 248864 | 248585 | 218220 | 200638 |
| Algae_Con_Dobu_67_21_L001 | 279757 | 270743 | 267826 | 267367 | 237707 | 208402 |
| Algae_Con_Illi_122_31_L001 | 245252 | 237348 | 233880 | 234254 | 204722 | 187467 |
| Algae_Con_Illi_195_29_L001 | 231458 | 225375 | 223788 | 223574 | 206900 | 188516 |
| Algae_Con_Illi_217_41_L001 | 236342 | 230287 | 228864 | 228658 | 214987 | 200468 |
| Algae_Con_Illi_219_36_L001 | 197659 | 193162 | 191760 | 191785 | 179776 | 168148 |
| Algae_Low_Dobu_14_16_L001 | 222918 | 215365 | 214913 | 214937 | 205754 | 199222 |
| Algae_Low_Dobu_97_39_L001 | 166469 | 163422 | 162260 | 162341 | 152081 | 133301 |
| Algae_Low_Dobu_98_6_L001 | 253389 | 245319 | 243137 | 242750 | 220384 | 190816 |
| Algae_Low_Illi_136_14_L001 | 204846 | 199244 | 198222 | 198347 | 184761 | 175278 |
| Algae_Low_illi_147_37_L001 | 175940 | 171900 | 171141 | 171286 | 164375 | 154247 |
| Algae_Low_Illi_176_18_L001 | 208290 | 203860 | 202536 | 202328 | 190742 | 171474 |
| Algae_Med_Dobu_76_28_L001 | 251984 | 245499 | 243779 | 243488 | 225827 | 199808 |
| Algae_Med_Dobu_91_24_L001 | 204713 | 200501 | 199694 | 199417 | 190485 | 164522 |
| Algae_Med_Dobu_92_11_L001 | 223704 | 218778 | 217381 | 217271 | 202034 | 180283 |
| Algae_Med_Illi_160_4_L001 | 252232 | 244127 | 242699 | 242472 | 223965 | 191434 |
| Algae_Med_Illi_229_27_L001 | 265603 | 254828 | 253113 | 252817 | 231272 | 197025 |
| Algae_Med_Illi_238_15_L001 | 227256 | 222078 | 221337 | 221262 | 212516 | 203184 |
| CS_Con_Illi_115_35_L001 | 179809 | 173064 | 172350 | 172412 | 167455 | 148847 |
| CS_Con_Illi_118_19_L001 | 144092 | 138597 | 137934 | 138064 | 132092 | 120691 |
| CS_Con_Illi_181_13_L001 | 188669 | 182150 | 181326 | 181278 | 171977 | 153743 |
| CS_Con_Illi_188_17_L001 | 197737 | 190966 | 190234 | 190222 | 181697 | 159505 |
| CS_Con_Illi_189_38_L001 | 222613 | 214665 | 213655 | 213638 | 202729 | 179048 |
| CS_Low_Illi_125_8_L001 | 214528 | 207111 | 206674 | 206542 | 199891 | 176112 |
| CS_Low_Illi_127_42_L001 | 86374 | 84623 | 83971 | 84073 | 80921 | 73982 |
| CS_Low_Illi_131_3_L001 | 199000 | 192488 | 191693 | 191742 | 184337 | 170098 |
| CS_Low_Illi_138_2_32_L001 | 224069 | 215075 | 214331 | 214369 | 204623 | 183065 |
| CS_Low_Illi_163_22_L001 | 231384 | 222240 | 220879 | 220871 | 208104 | 184957 |
| CS_Low_Illi_173_23_L001 | 229868 | 219886 | 219057 | 219051 | 208308 | 176933 |
| CS_Low_Illi_179_33_L001 | 193036 | 186030 | 185516 | 185530 | 177605 | 154953 |
| CS_Med_Illi_149_30_L001 | 183699 | 178306 | 177506 | 177563 | 171020 | 155534 |
| CS_Med_Illi_159_43_L001 | 167353 | 163555 | 162763 | 162855 | 156836 | 140975 |
| CS_Med_Illi_216_5_L001 | 225210 | 215461 | 214099 | 213825 | 200874 | 184990 |
| CS_Med_Illi_227_7_L001 | 238422 | 229429 | 228004 | 228047 | 214483 | 196621 |
| CS_Med_Illi_234_1_L001 | 197808 | 190576 | 189805 | 189815 | 181772 | 156451 |
| PNG_100_13_11_L001 | 330589 | 311170 | 304494 | 305699 | 250398 | 234943 |
| PNG_100_14_5_L001 | 314877 | 303858 | 297306 | 298246 | 246814 | 234403 |
| PNG_100_15_8_L001 | 289767 | 280567 | 273938 | 274845 | 219173 | 204595 |
| PNG_100_16_21_L001 | 345584 | 329523 | 320143 | 322110 | 255499 | 242937 |
| PNG_100_17_13_L001 | 286945 | 276639 | 269077 | 270403 | 217629 | 207103 |
| PNG_100_18_6_L001 | 338835 | 323266 | 315339 | 316767 | 254121 | 240073 |
| PNG_100_19_14_L001 | 319513 | 307144 | 299205 | 300686 | 238634 | 226747 |
| PNG_100_20.2_23_L001 | 259250 | 250388 | 244419 | 245958 | 197184 | 186771 |
| PNG_100_21_7_L001 | 299573 | 287304 | 279430 | 281064 | 221476 | 207330 |
| PNG_100_22_24_L001 | 309874 | 299061 | 290381 | 292163 | 226277 | 212596 |
| PNG_100_23_9_L001 | 333635 | 319618 | 311372 | 313253 | 253262 | 240436 |
| PNG_100_24_33_L001 | 253625 | 244651 | 239091 | 240459 | 194681 | 185195 |
| PNG_100_26_2_L001 | 350935 | 335928 | 327739 | 329073 | 265312 | 253694 |
| PNG_100_27_17_L001 | 307495 | 296167 | 289194 | 290705 | 237821 | 228390 |
| PNG_100_28_18_L001 | 298447 | 286205 | 278163 | 279792 | 219357 | 209516 |
| PNG_100_29_32_L001 | 359480 | 345972 | 336824 | 339063 | 269290 | 257950 |
| PNG_100_30_30_L001 | 323027 | 301120 | 292785 | 294861 | 231329 | 219863 |
| PNG_Sessile_13_20_L001 | 240974 | 229860 | 226959 | 227087 | 199692 | 187083 |
| PNG_Sessile_14_3_L001 | 300629 | 284978 | 281939 | 281608 | 250191 | 233314 |
| PNG_Sessile_16_1_L001 | 353218 | 338027 | 332917 | 333120 | 290951 | 277508 |
| PNG_Sessile_17_22_L001 | 282939 | 271394 | 268660 | 268531 | 245572 | 239446 |
| PNG_Sessile_18_19_L001 | 238536 | 228719 | 225957 | 225935 | 200762 | 191858 |
| PNG_Sessile_21_12_L001 | 309129 | 294873 | 290041 | 290563 | 244201 | 229676 |
| PNG_Sessile_22_4_L001 | 502224 | 484143 | 478318 | 478372 | 429054 | 409784 |
| PNG_Sessile_23_28_L001 | 256260 | 245311 | 242386 | 242826 | 216375 | 202197 |
| PNG_Sessile_24_31_L001 | 173125 | 166005 | 164564 | 164246 | 150641 | 137622 |
| PNG_Sessile_25_10_L001 | 224435 | 218903 | 216195 | 216750 | 193250 | 183066 |
| PNG_Sessile_26_16_L001 | 244837 | 234790 | 231771 | 232017 | 207985 | 200470 |
| PNG_Sessile_27_15_L001 | 333001 | 319215 | 316125 | 316248 | 288527 | 277794 |
| PNG_Sessile_28_29_L001 | 309011 | 293688 | 290366 | 290464 | 262906 | 255078 |
| PNG_Sessile_29_26_L001 | 284053 | 271479 | 267879 | 268231 | 233438 | 215500 |
| PNG_Sessile_30_27_L001 | 286848 | 274801 | 271563 | 271948 | 244209 | 228885 |
| RVS_Con_Dobu_106_29_L001 | 255691 | 249711 | 247468 | 247958 | 226152 | 208667 |
| RVS_Con_Dobu_49_1_L001 | 312551 | 306940 | 304427 | 304886 | 284364 | 264561 |
| RVS_Con_Dobu_50_12_L001 | 245596 | 239837 | 238335 | 238748 | 219352 | 203899 |
| RVS_Con_Dobu_56_14_L001 | 322447 | 315942 | 313821 | 314227 | 294940 | 279558 |
| RVS_Con_Dobu_66_11_L001 | 249107 | 244906 | 243804 | 243824 | 239023 | 224668 |
| RVS_Con_Illi_182_12_L001 | 442458 | 428011 | 425893 | 425919 | 407850 | 356948 |
| RVS_Con_Illi_187_9_L001 | 214787 | 209701 | 209039 | 209232 | 205095 | 183586 |
| RVS_Con_Illi_191_6_L001 | 209585 | 205367 | 203926 | 204264 | 192499 | 175752 |
| RVS_Con_Illi_192_34_L001 | 172815 | 168961 | 168246 | 168428 | 164487 | 155304 |
| RVS_Con_Illi_197_5_L001 | 247335 | 242621 | 242058 | 242162 | 219376 | 212516 |
| RVS_Con_Illi_207_2_L001 | 294571 | 287694 | 287112 | 287040 | 263528 | 245683 |
| RVS_Con_Illi_220_20_L001 | 210563 | 203970 | 202759 | 202834 | 190653 | 172255 |
| RVS_Low_Dobu_11_8_L001 | 190947 | 187452 | 186913 | 187004 | 180128 | 155890 |
| RVS_Low_Dobu_18_9_L001 | 185712 | 181371 | 180632 | 180808 | 174938 | 157392 |
| RVS_Low_Dobu_3_21_L001 | 204750 | 200014 | 199438 | 199621 | 193927 | 176539 |
| RVS_Low_Dobu_8_10_L001 | 237298 | 232840 | 232398 | 232565 | 230242 | 211504 |
| RVS_Low_Illi_123_10_L001 | 146872 | 143392 | 142706 | 142864 | 136554 | 129585 |
| RVS_Low_Illi_128_25_L001 | 204706 | 198998 | 197732 | 197995 | 190665 | 179733 |
| RVS_Low_Illi_140_20_L001 | 274878 | 267289 | 265399 | 265420 | 220332 | 199967 |
| RVS_Low_Illi_167_27_L001 | 261441 | 253941 | 251980 | 252397 | 226366 | 210432 |
| RVS_Low_Illi_168_3_L001 | 259359 | 253267 | 252278 | 252503 | 222379 | 208854 |
| RVS_Med_Dobu_68_22_L001 | 282668 | 276719 | 275292 | 275470 | 251739 | 236006 |
| RVS_Med_Dobu_69_23_L001 | 278873 | 274430 | 273438 | 273561 | 244971 | 228638 |
| RVS_Med_Dobu_74_24_L001 | 222281 | 216734 | 214896 | 215265 | 193547 | 177740 |
| RVS_Med_Dobu_81_13_L001 | 278193 | 271775 | 270594 | 270654 | 261955 | 235400 |
| RVS_Med_Dobu_82_15_L001 | 232768 | 227776 | 226690 | 226819 | 219589 | 199053 |
| RVS_Med_Illi_153_2_L001 | 183303 | 176642 | 176295 | 176303 | 171132 | 154068 |
| RVS_Med_Illi_157_4_L001 | 301096 | 294819 | 293202 | 293584 | 276353 | 257027 |
| RVS_Med_Illi_226_25_L001 | 250046 | 243319 | 242172 | 242270 | 185221 | 172907 |
| RVS_Med_Illi_235_17_L001 | 244998 | 238608 | 237247 | 237595 | 213937 | 191960 |

**23S gene rRNA Samples**

| 103_S18_L001 | 781490 | 546317 | 544133 | 545420 | 487187 | 412556 |
| --- | --- | --- | --- | --- | --- | --- |
| 104_S1_L001 | 926162 | 652873 | 647090 | 650839 | 535838 | 358574 |
| 122_S2_L001 | 795286 | 663331 | 660434 | 662099 | 599077 | 440501 |
| 136_S7_L001 | 551363 | 464795 | 464558 | 464721 | 456791 | 424202 |
| 14_S16_L001 | 745075 | 640340 | 640212 | 639271 | 633178 | 630362 |
| 147_S15_L001 | 812840 | 692932 | 692785 | 692784 | 685455 | 665890 |
| 160_S5_L001 | 867809 | 721610 | 719964 | 720896 | 678427 | 491319 |
| 176_S6_L001 | 818234 | 647976 | 645402 | 646265 | 591886 | 426523 |
| 195_S9_L001 | 801525 | 624058 | 620092 | 622322 | 541135 | 343497 |
| 217_S10_L001 | 956026 | 734616 | 732236 | 733466 | 684859 | 544089 |
| 219_S4_L001 | 657509 | 466626 | 464740 | 465514 | 422984 | 324689 |
| 229_S3_L001 | 550697 | 447842 | 445454 | 447115 | 401876 | 259798 |
| 238_S19_L001 | 770953 | 669425 | 669162 | 669316 | 662894 | 615478 |
| 67_S17_L001 | 956712 | 686628 | 683695 | 685376 | 627183 | 513594 |
| 76_S11_L001 | 595232 | 425506 | 424761 | 425158 | 390968 | 293756 |
| 91_S12_L001 | 716419 | 605092 | 604688 | 604653 | 586314 | 530722 |
| 92_S8_L001 | 787592 | 645308 | 643070 | 644527 | 598457 | 404055 |
| 97_S14_L001 | 542006 | 405464 | 405221 | 405305 | 393478 | 366534 |
| 98_S13_L001 | 823113 | 668455 | 667305 | 667722 | 631826 | 524127 |
| Undetermined_S0_L001 | 7707066 | 672966 | 667880 | 668943 | 532745 | 429654 |

**Table S2:** Effect size and significance of factors in PERMANOVAs describing the compositional differences among sites, and along the pH gradient for all multiomic data types; site fit as a blocking factor, except in the case of *Halisarca* sp. sponge fractions, which were only collected at one site. A p-value less than 0.05 indicates that the factor significantly affects composition. Bonferroni correction was applied to all PERMANOVA.

***Sequence PERMANOVA***

| A) Benthic Photosynthetic Community  Microbiome (16S gene rRNA) | Pseudo-F | p-value |
| --- | --- | --- |
| Locality | 2.3 | **0.05** |
| pH | 6.2 | **0.05** |

| B) Sediment  Microbiome (16S gene rRNA) | Pseudo-F | P value |
| --- | --- | --- |
| Locality | 3.9 | **0.01** |
| pH | 3.9 | **0.01** |

| C) Benthic Holobiont Community  Microbiome (16S gene rRNA) | Pseudo-F | P value |
| --- | --- | --- |
| Locality | 3.0 | **0.04** |
| pH | 4.5 | **0.04** |

| D) *Halisarca* Sponge Microbiome (16S gene rRNA) | Pseudo-F | P value |
| --- | --- | --- |
| pH | 0.28 | 1 |

| E) *Tethya* Sponge Microbiome (16S gene rRNA) | Pseudo-F | P value |
| --- | --- | --- |
| Locality | 3.7 | **0.03** |
| pH | 5.9 | **0.03** |

***Metabolome PERMANOVA***

| F) Benthic Photosynthetic Community Metabolome | Pseudo-F | P value |
| --- | --- | --- |
| Locality | 1.3 | 1 |
| pH | 0.8 | 1 |

| G) Sediment Metabolome | Pseudo-F | P value |
| --- | --- | --- |
| Locality | 3.1 | 1 |
| pH | 1.4 | 1 |

| H) Benthic Holobiont Community Metabolome | Pseudo-F | P value |
| --- | --- | --- |
| Locality | 0.48 | 1 |
| pH | 0.73 | 1 |

| I) *Halisarca* Sponge Metabolome | Pseudo-F | P value |
| --- | --- | --- |
| pH | 0.95 | 1 |

| J) *Tethya* Sponge Metabolome | Pseudo-F | P value |
| --- | --- | --- |
| Locality | 0.37 | 1 |
| pH | 1.2 | 1 |

**Table S3:** ASV estimated richness betta mixed model: fixed effects listed, random effect of locality. Model Explanatory Power: test statistic = 117.2, p<0.05. A p-value less than 0.05 indicates that the explanatory variable significantly affects ASV richness. All values in the table are reported to two significant figures.

| Variable Category | Explanatory variable | Estimate | Standard error | p-value |
| --- | --- | --- | --- | --- |
| Intercept | Intercept (Sediment Microbiome) | 12000 | 500 | **<0.001** |
| Community | Benthic Holobiont Community Microbiome | -3800 | 1500 | **0.01** |
|  | Benthic Photosynthetic Community Microbiome | -9100 | 1500 | **<0.001** |
|  | *Halisarca* Sponge Microbiome | -10000 | 1400 | **<0.001** |
|  | *Tethya* Sponge Microbiome | -9700 | 1000 | **<0.001** |
| pH | pH | -260 | 990 | 0.80 |
| Community : pH Interactions | Benthic Holobiont Community Microbiome : pH | -980 | 2500 | 0.69 |
|  | Benthic Photosynthetic Community Microbiome : pH | -1500 | 2600 | 0.56 |
|  | *Halisarca* Sponge Microbiome : pH | 220 | 2200 | 0.92 |
|  | *Tethya* Sponge Microbiome : pH | 62 | 1800 | 0.97 |

**Table S4:** Phylum Shannon diversity betta mixed model: fixed effects listed, random effect of locality. Model Explanatory Power: test statistic = 3197.94, p<0.05. A p-value less than 0.05 indicates that the explanatory variable significantly affects metabolite richness. All values in the table are reported to two significant figures.

| Variable Category | Explanatory variable | Estimate | Standard error | p-value |
| --- | --- | --- | --- | --- |
| Intercept | Intercept (Sediment Microbiome) | 2.1 | 0.027 | **<0.001** |
| Community | Benthic Holobiont Community Microbiome | -0.13 | 0.067 | 0.06 |
|  | Benthic Photosynthetic Community Microbiome | -0.74 | 0.067 | **<0.001** |
|  | *Halisarca* Sponge Microbiome | -1.2 | 0.02 | **<0.001** |
|  | *Tethya* Sponge Microbiome | -1.2 | 0.048 | **<0.001** |
| pH | pH | -0.018 | 0.045 | 0.69 |
| Community : pH Interactions | Benthic Holobiont Community Microbiome : pH | 0.4 | 0.11 | 0.71 |
|  | Benthic Photosynthetic Community Microbiome : pH | -0.32 | 0.12 | **0.006** |
|  | *Halisarca* Sponge Microbiome : pH | 0.051 | 0.10 | 0.61 |
|  | *Tethya* Sponge Microbiome : pH | -0.28 | 0.081 | **0.001** |

**Table S5:** ASV Shannon diversity betta mixed model: fixed effects listed, random effect of locality. R Squared (conditional)= 88.8%. A p-value less than 0.05 indicates that the explanatory variable significantly affects metabolite richness. All values in the table are reported to two significant figures.

| Variable Category | Explanatory variable | Estimate | Standard error | p-value |
| --- | --- | --- | --- | --- |
| Intercept | Intercept (Sediment Microbiome) | 8.1 | 0.18 | **<0.001** |
| Community | Benthic Holobiont Community Microbiome | -1.6 | 0.28 | **<0.001** |
|  | Benthic Photosynthetic Community Microbiome | -4.0 | 0.28 | **<0.001** |
|  | *Halisarca* Sponge Microbiome | -4.7 | 0.027 | **<0.001** |
|  | *Tethya* Sponge Microbiome | -5.6 | 0.24 | **<0.001** |
| pH | pH | -0.23 | 0.32 | 0.48 |
| Community : pH Interactions | Benthic Holobiont Community Microbiome : pH | -0.03 | 0.46 | 0.94 |
|  | Benthic Photosynthetic Community Microbiome : pH | -1.6 | 0.47 | **0.001** |
|  | *Halisarca* Sponge Microbiome : pH | 0.15 | 0.44 | 0.74 |
|  | *Tethya* Sponge Microbiome : pH | -0.06 | 0.40 | 0.87 |

**Table S6:** Summary of tests and results for 16S rRNA gene and metabolomic data

|  | **Composition** | **Richness** | **Shannon diversity (ASV)** | **Shannon diversity (phyla)** | **Multiomic distinctness** |
| --- | --- | --- | --- | --- | --- |
| Benthic Photosynthetic Community microbiome | Community shift | - | Declined with OA | Declined with OA | NA |
| Sediment microbiome | Community shift | - | - | - | NA |
| Benthic Holobiont community microbiome | Community shift | - | - | - | Declined with OA |
| *Halisarca* sp. sponge microbiome | - | - | - | - | Declined with OA |
| *Tethya* sp. sponge microbiome | Community shift | - | - | Declined with OA |  |
| Benthic Photosynthetic Community metabolome | - | - | - | | NA |
| Sediment metabolome | - | - | Increased with OA | | NA |
| Benthic Holobiont Community metabolome | - | - | Declined with OA | | Declined with OA |
| *Halisarca* sp. sponge metabolome | - | - | - | | - |
| *Tethya* sp. sponge metabolome | - | - | - | | - |

**Table S7:** Compound richness linear mixed model: fixed effects listed, random effect of locality. R Squared (conditional)= 24.5%. A p-value less than 0.05 indicates that the explanatory variable significantly affects ASV richness. All values in the table are reported to two significant figures.

| Variable Category | Explanatory variable | Estimate | Standard error | p-value |
| --- | --- | --- | --- | --- |
| Intercept | Intercept (Sediment Metabolome) | 170. | 10 | **<0.001** |
| Community | Benthic Holobiont Community Metabolome | 19. | 14 | 0.18 |
|  | Benthic Photosynthetic Community Metabolome | -2.5 | 14 | 0.86 |
|  | *Halisarca* Sponge Metabolome | -31 | 14 | **0.03** |
|  | *Tethya* Sponge Metabolome | 31 | 13 | **0.01** |
| pH | pH | 24 | 17 | 0.18 |
| Community : pH Interactions | Benthic Holobiont Community Metabolome : pH | -49 | 25 | 0.051 |
|  | Benthic Photosynthetic Community Metabolome : pH | -6.6 | 24 | 0.79 |
|  | *Halisarca* Sponge Metabolome : pH | -12 | 25 | 0.62 |
|  | *Tethya* Sponge Metabolome | -6.7 | 21 | 0.76 |

**Table S8:** Compound Shannon diversity linear mixed model: fixed effects listed, random effect of locality. R Squared (conditional)= 32.0%. A p-value less than 0.05 indicates that the explanatory variable significantly affects ASV richness. All values in the table are reported to two significant figures.

| Variable Category | Explanatory variable | Estimate | Standard error | p-value |
| --- | --- | --- | --- | --- |
| Intercept | Intercept (Sediment Metabolome) | 2.76 | 0.13 | **<0.001** |
| Community | Benthic Holobiont Community Metabolome | 0.04 | 0.16 | 0.79 |
|  | Benthic Photosynthetic Community Metabolome | 0.07 | 0.16 | 0.65 |
|  | *Halisarca* Sponge Metabolome | -0.27 | 0.17 | 0.11 |
|  | *Tethya* Sponge Metabolome | 0.52 | 0.14 | **<0.001** |
| pH | pH | 0.41 | 0.2 | **0.04** |
| Community : pH Interactions | Benthic Holobiont Community Metabolome : pH | -0.63 | 0.28 | **0.03** |
|  | Benthic Photosynthetic Community Metabolome : pH | -0.27 | 0.28 | 0.34 |
|  | *Halisarca* Sponge Metabolome : pH | -0.12 | 0.28 | 0.69 |
|  | *Tethya* Sponge Metabolome | -0.22 | 0.25 | 0.36 |

## **Supplementary Figures**


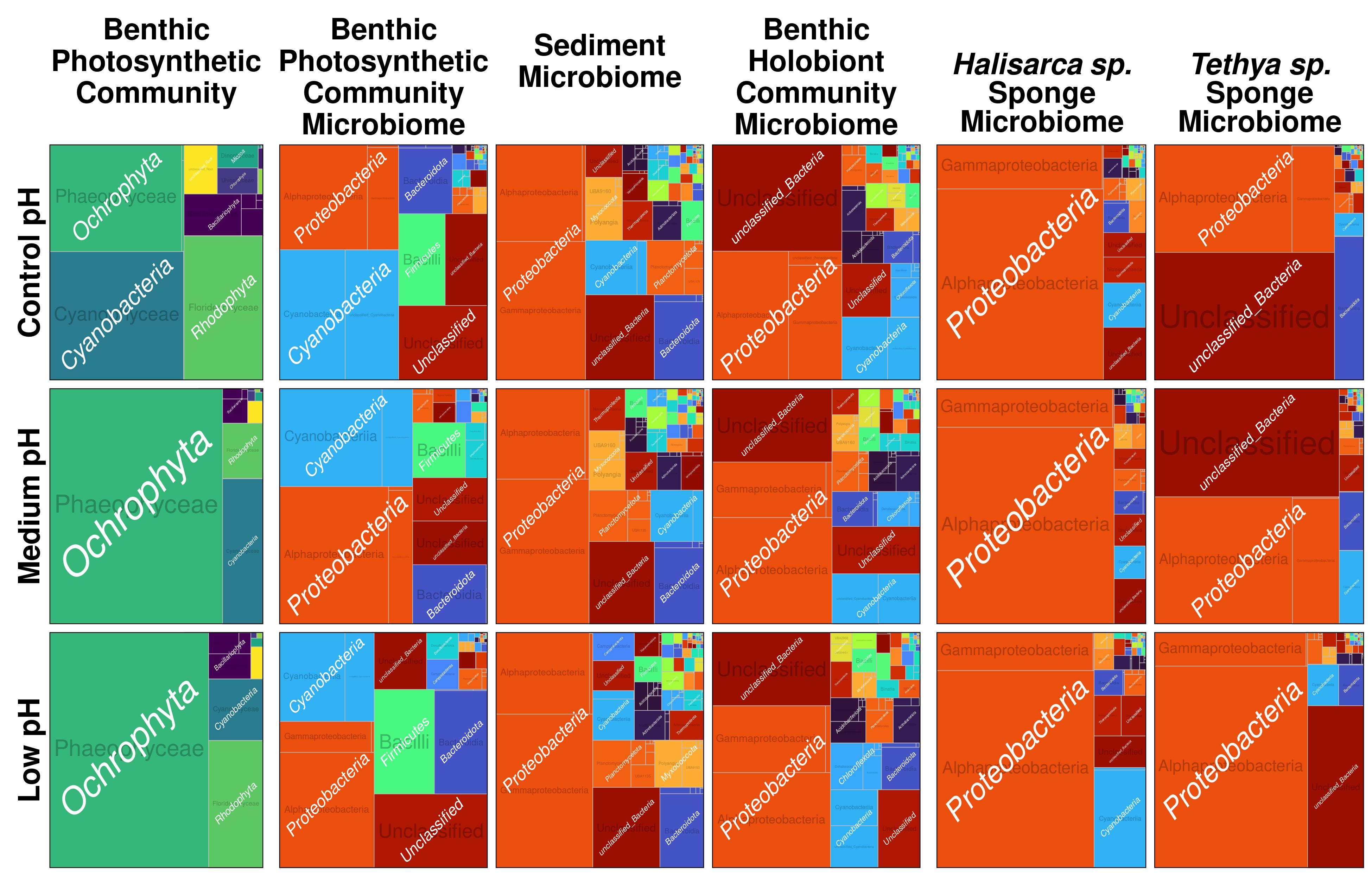
**Figure S1:** Visual representation of read abundance from the ARMS 23S rRNA gene and 16S rRNA gene metabarcoding dataset. Data are aggregated across biological replicates to present the average composition of each fraction, at each pH, showing phylum (in white text) and class (in grey text).


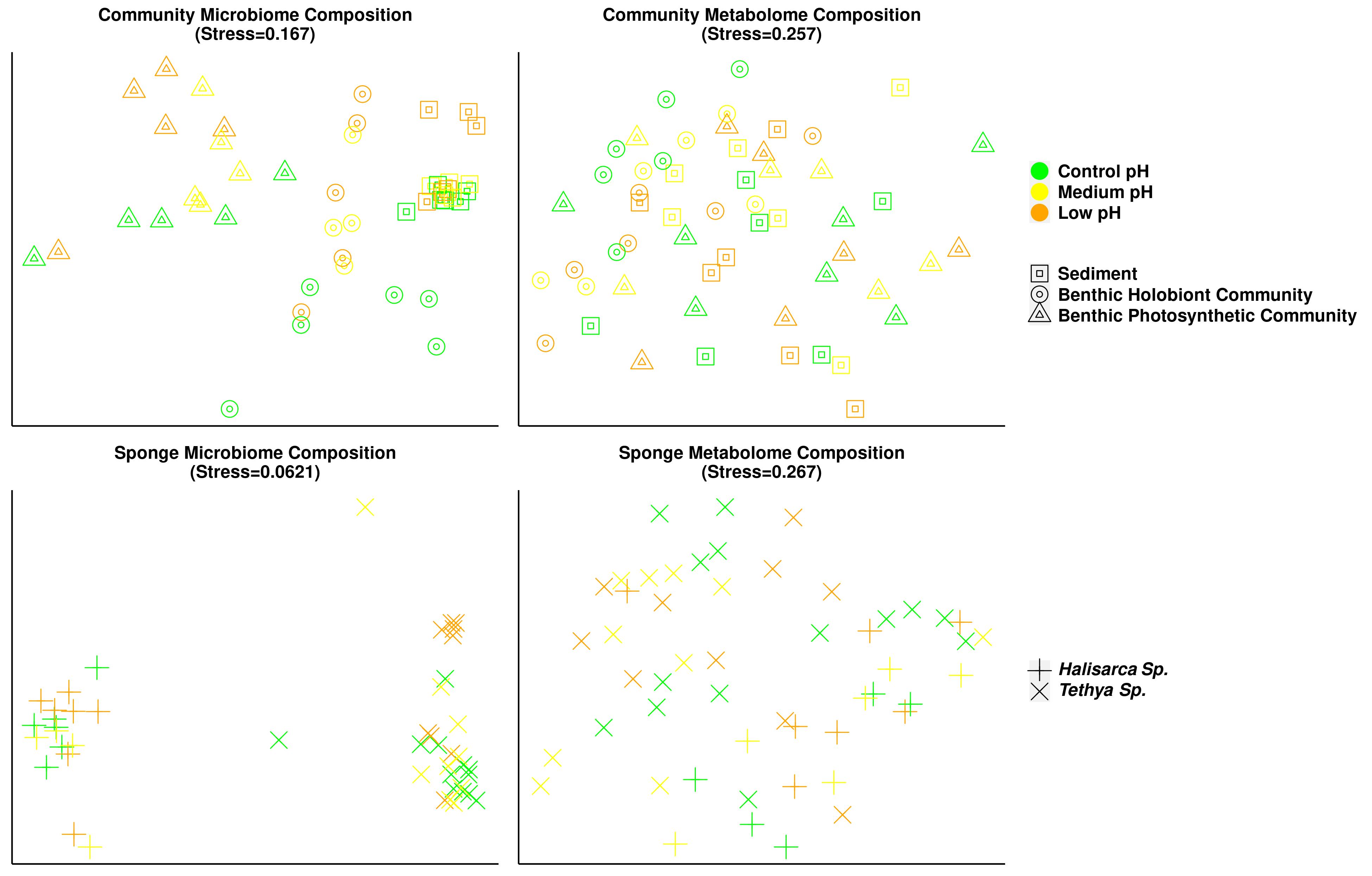
**Figure S2:** NMDS of microbiome and metabolome composition of all fractions across the pH gradient, calculated using Morisita dissimilarity.


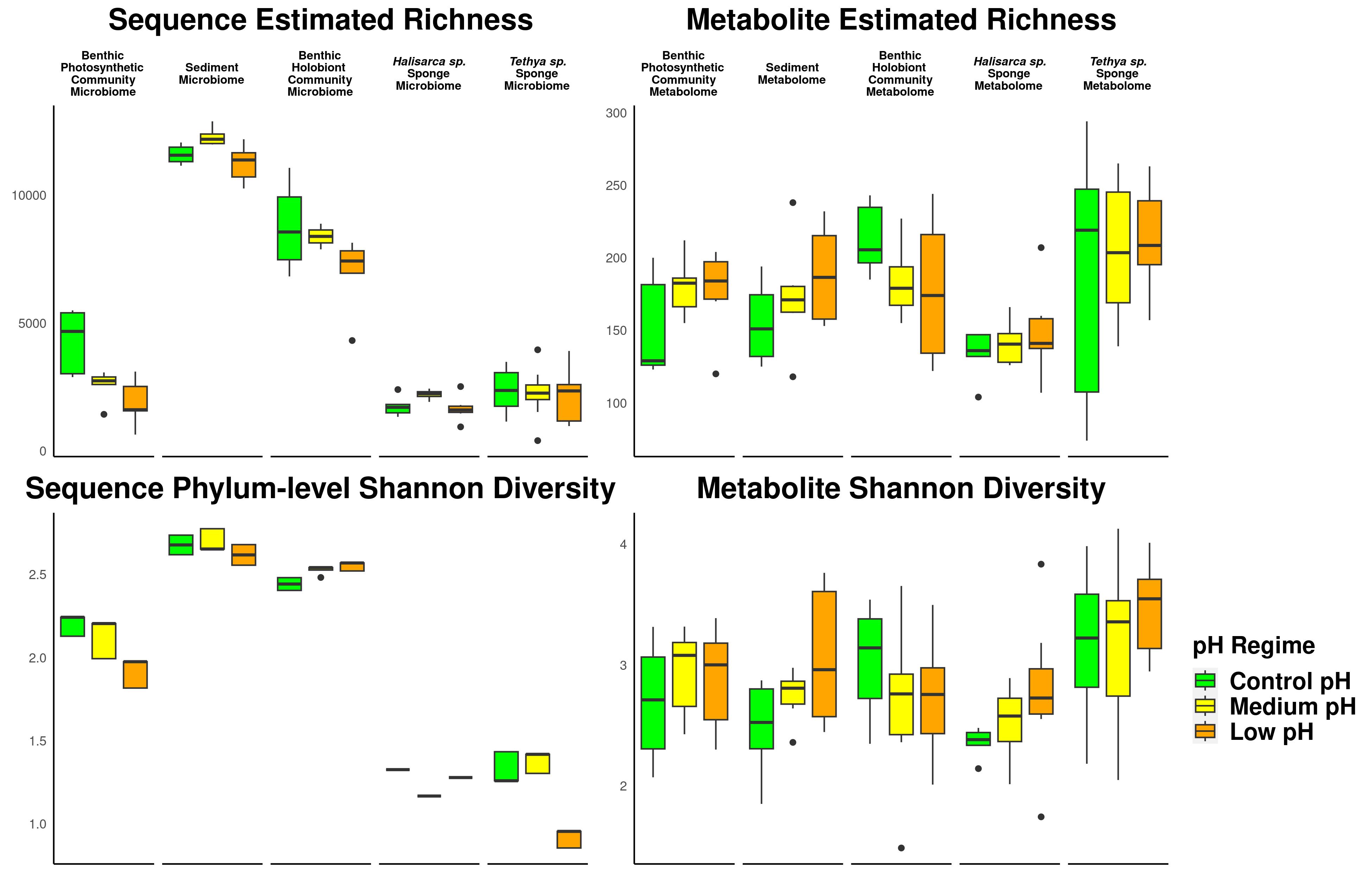
**Figure S3**: Microbial and chemical richness and Shannon diversity boxplots for all fractions across the pH gradient.
